# Designer Gelators for the Crystallization of a Salt Active Pharmaceutical Ingredient—Mexiletine Hydrochloride

**DOI:** 10.1021/acs.cgd.2c00925

**Published:** 2022-10-12

**Authors:** Jessica L. Andrews, Stuart R. Kennedy, Dmitry S. Yufit, James F. McCabe, Jonathan W. Steed

**Affiliations:** † Department of Chemistry, Durham University, South Road, Durham DH1 3LE, U.K.; ‡ Pharmaceutical Sciences, R&D, AstraZeneca, Charter Way, Silk Road Business Park, Macclesfield SK10 2NA, U.K.

## Abstract

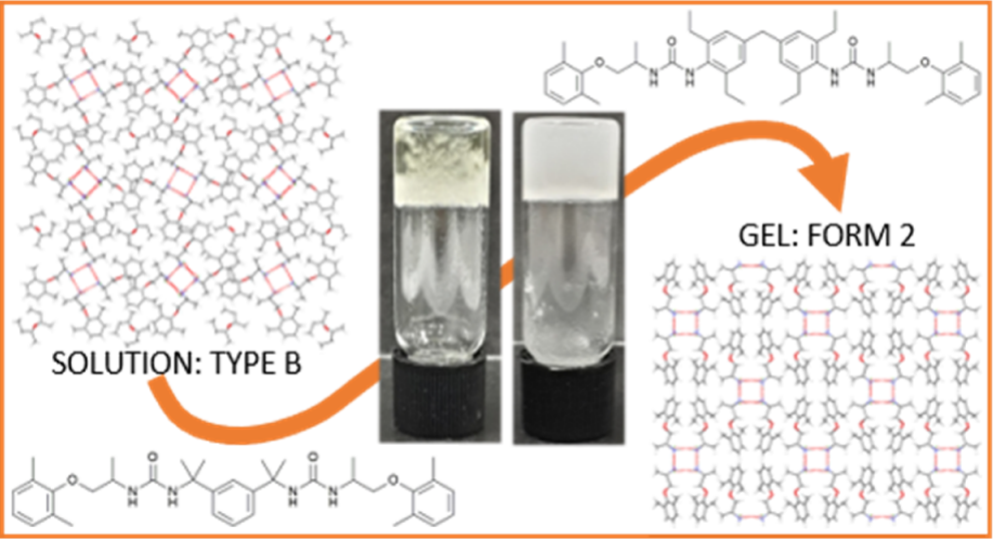

We report an approach to obtain drug-mimetic supramolecular gelators, which are capable of stabilizing metastable polymorphs of the pharmaceutical salt mexiletine hydrochloride, a highly polymorphic antiarrhythmic drug. Solution-phase screening led to the discovery of two new solvated solid forms of mexiletine, a type C 1,2,4-trichlorobenzene tetarto-solvate and a type D nitrobenzene solvate. Various metastable forms were crystallized within the gels under conditions which would not have been possible in solution. Despite typically crystallizing concomitantly with form 1, a pure sample of form 3 was crystallized within a gel of ethyl methyl ketone. Various type A channel solvates were crystallized from gels of toluene and ethyl acetate, in which the contents of the channels varied from those of solution-phase forms. Most strikingly, the high-temperature-stable form 2 was crystallized from a gel in 1,2-dibromoethane: the only known route to access this form at room temperature. These results exemplify the powerful stabilizing effect of drug-mimetic supramolecular gels, which can be exploited in pharmaceutical polymorph screens to access highly metastable or difficult-to-nucleate solid forms.

## Abstract 2

Tailored supramolecular gels stabilize the metastable polymorphs of mexiletine hydrochloride. Selective nucleation of various high-energy solid forms was observed within the gel environment. Most notably, the high-temperature-stable form 2 was crystallized from a gel at room temperature, showing the powerful stabilizing effect of drug-mimetic supramolecular gels.

## Introduction

The polymorphs of an active pharmaceutical ingredient (API) can have drastically different physical and chemical properties, which change the way that drug molecules are released from the formulation and absorbed into the blood stream of a patient.^1^ The emergence of a late-appearing, more stable solid form can significantly impact the processing, manufacture, storage, administration, and efficacy of the drug, leading to high reformulation costs for the manufacturer.^2−5^ Therefore, controlling the solid form of an API is of paramount importance in creating a safe and effective medicine, and there is a strong motivation for pharmaceutical companies to thoroughly characterize the solid-form landscape of new APIs.^6,7^ As a result, many novel crystallization techniques have emerged to increase the scope of traditional solution-phase crystallization approaches and ensure the solid-form landscape of an API is fully understood before formulating and marketing the product. These techniques also reduce the gap between the large range of solid forms often revealed by computational crystal structure calculation approaches and the smaller number realized experimentally.^8,9^ Novel crystallization techniques include the use of soluble crystallization additives, heterogeneous nucleation, epitaxy, macro- and nano-scale confinement, nanodroplet crystallization, microemulsions, self-assembled monolayers, and gel-phase crystallization.^10−16^ Gel-phase crystallization originated from the field of protein crystallography, in which polymeric hydrogels such as silica or agarose are used to increase the crystal quality by slowing the diffusion and limiting the nucleation.^17−19^ Small-molecule supramolecular gels, held together by non-covalent interactions, are tunable, reversible, and more varied in structure than their polymeric counterparts.^20−24^ Several studies report alterations to the size, habit (external shape), quality, and solid form of crystals grown within supramolecular gels. In some cases, the self-assembly processes of the gel and crystals are orthogonal, and changes in the solid form are derived from reduced nucleation within the gel environment.^12,25−27^ Whereas, in others, gelators have been designed to interact with a target drug. In these systems, the gel fibers can act as a heterogeneous nucleation surface and provide a template to encourage epitaxial overgrowth of highly metastable or difficult-to-nucleate solid forms, and/or serve to bias the conformational distribution during nucleation.^28−33^ If the correct functionality is included, the gelation can even be switched off by the addition of anions, so that the crystals can be retrieved by filtration.^34^

Gelator molecules can interact with a crystallizing drug by several different mechanisms. Acid-amine hydrogen bonds between a carboxylic acid-containing drug and an amine-containing dendron gelator have supported the crystallization of an unusual polymorph of carbamazepine.^30^ Similarly, chlorphenesin was crystallized from a calixarene-based gel, in which the drug molecules are included within the hydrophobic cavities along the gel fibers.^31^ Cisplatin-mimicking gelators have shown that incorporating some chemical functionality from the drug structure into the gelator provides a template for the crystallization of unusual drug solid forms; in this case, a previously unknown solvate of cisplatin.^32^ Similarly, ROY-mimetic gelators containing the same torsion angle as ROY’s metastable R polymorph led to the reliable crystallization of this form from solvents that would typically crystallize the thermodynamically stable Y form.^28^ A recent study suggests that, in systems where there is a significant interaction between the drug and the gelator, nucleation of the gel fibers and drug crystals can become competitive rather than orthogonal processes, preventing the formation of a gel network.^35^

**Figure 1:**
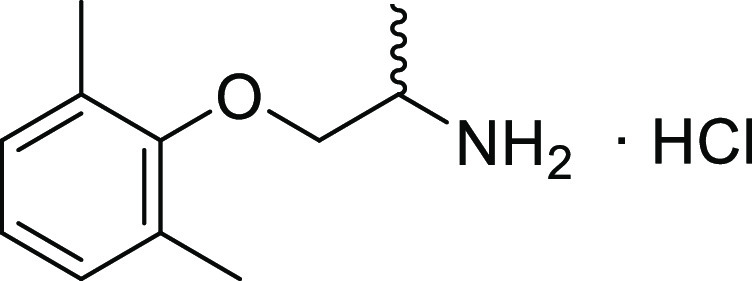
Structure of mexiletine hydrochloride.

This work reports the design of drug-mimetic supramolecular gelators for the crystallization of the antiarrhythmic drug salt, mexiletine hydrochloride (Figure 1). According to our previous work, mexiletine has five solid forms.^36^ Forms 1,^37^ 2, and 3^38^ are mutually enantiotropically related anhydrous polymorphs, with form 1 being the room-temperature-stable form, form 2 the high-temperature form, and form 3 the thermodynamically stable polymorph between 148 and 167 °C. There are two related families of metastable channel solvates termed type A and type B.

Mexiletine is a prolific solvate former, with 11 members of each family discovered to date. It is therefore likely that more solvated forms with a similar structure are possible. Several forms of mexiletine also crystallize as mixtures because two polymorphs are close together in energy. The potential for undiscovered solid forms and the opportunity to separate concomitantly crystallizing polymorphs make mexiletine HCl an ideal candidate for gel-phase crystallization. Indeed, gel-phase crystallization of mexiletine has already been attempted by our group, using a nanocellulose gelator that was designed to form hydrogen bonds with the target drugs. However, due to the high solubility of mexiletine in the solvent used to form these gels, the drug did not crystallize.^33^ The gelators described in the present work gelate a much wider range of solvents and therefore present a greater opportunity for drug crystallization. Moreover, mexiletine hydrochloride is a salt, and hydrogen bonding interactions with hydrogen bond acceptors such as chloride can inhibit gel formation. Indeed, crystallization of salt APIs from supramolecular gels has not yet been achieved.

## Gelator Design

**Figure 2:**
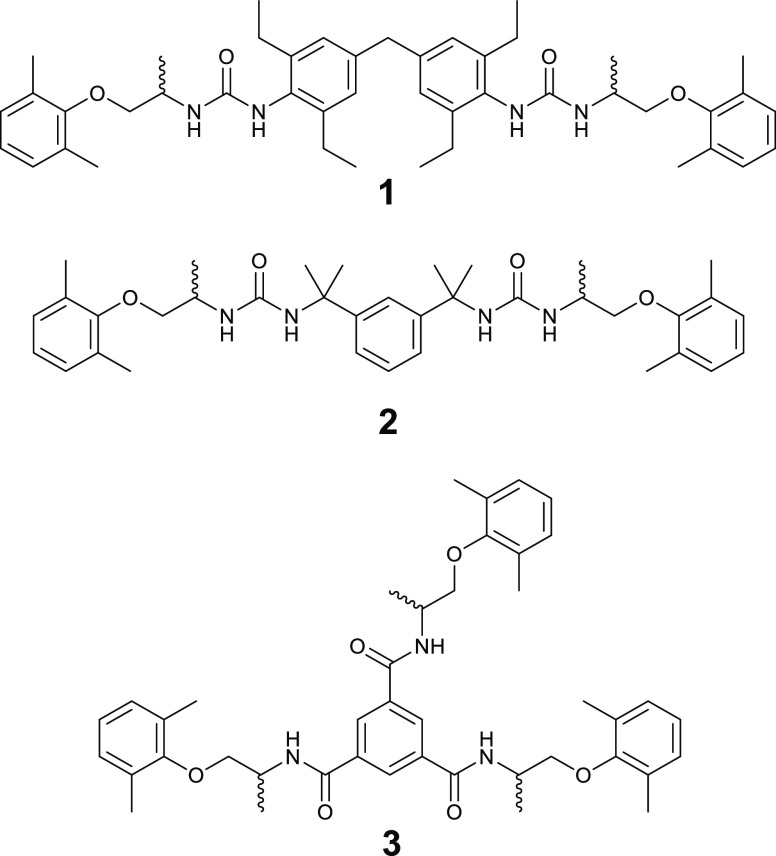
Structure of the three mexiletine mimetic gelators designed in the present study, compounds **1**, **2**, and **3**.

Two of the gelators used in this study (**1** and **2**) are bis(urea)s, composed of a central linking group that provides the gelling properties of the molecule, and mexiletine mimetic end groups that act as a template for the crystallizing drug. The linking groups were chosen due to their strong gelling tendency, which has been discussed widely in previous work from our group.^28,34,35,39−49^ We also prepared a tris(amide) gelator (**3**), with a central benzene 1,3,5-tricarboxamide group, derivatives of which have previously demonstrated reliable hydro-^50−53^ and organo-gelation^54−58^ behavior. The terminal amine in mexiletine HCl means that the entire drug structure can easily be connected to the linker molecule. Using the whole molecule as an end group, instead of mimicking one structural feature,^28,32,40^ is anticipated to strengthen the crystallization templating effect by increasing the structural similarity between the drug and the gelator.^40^ The three gelators used in this study are shown in Figure 2.

**Figure 3:**
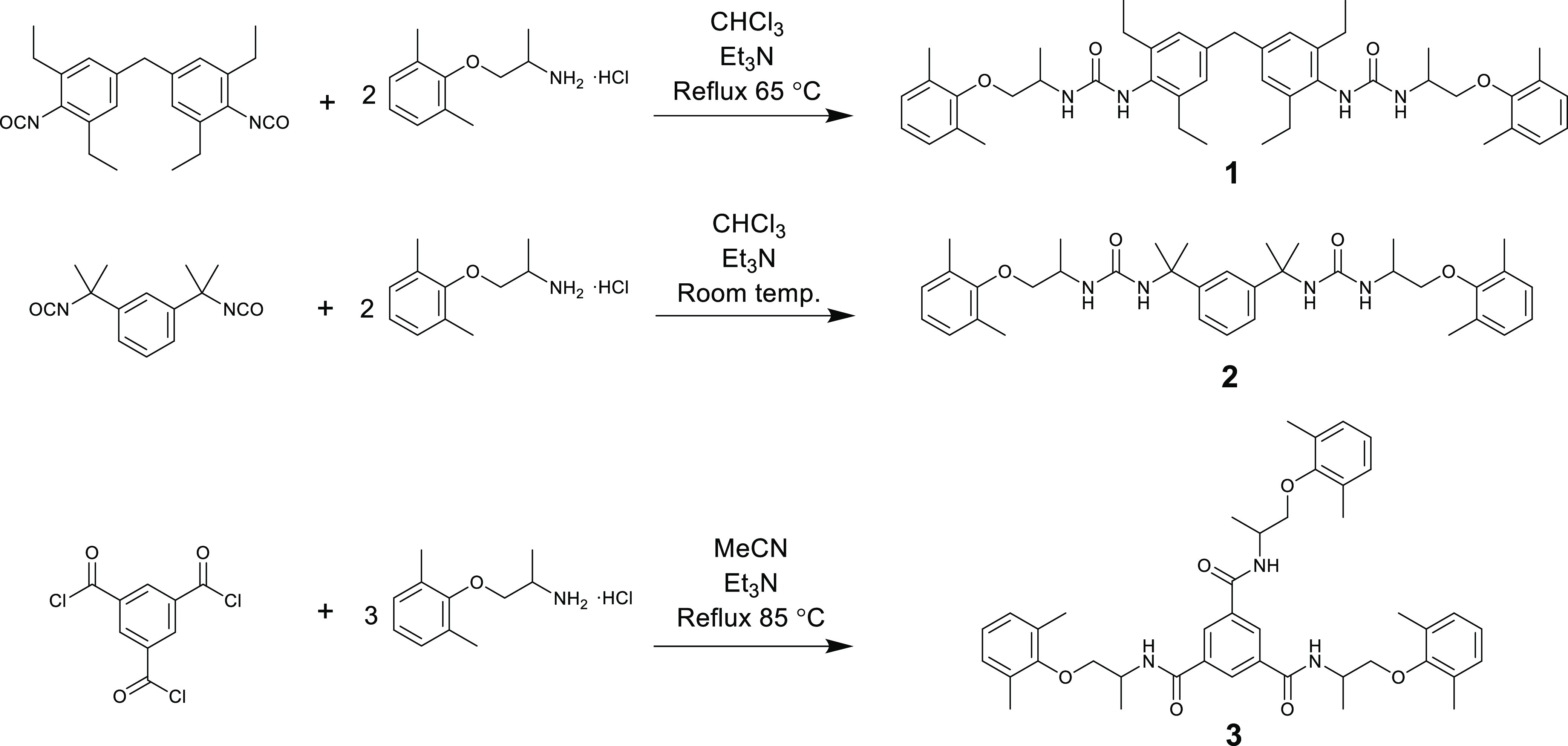
Synthetic routes to compounds **1**, **2**, and **3**.

All three gelators were synthesized using simple, one-step reactions between the linking group and mexiletine HCl, in the presence of triethylamine (Figure 3). In the bis(urea) syntheses, the isocyanate form of the linking group was used, whereas 1,3,5-benzenetricarbonyl trichloride was used to make the trisamide gelator. The isocyanate form of linker **1** was synthesized according to the literature method, from the corresponding amine and di-*tert*-butyl dicarbonate^59^ (Figure S1), whereas the other starting materials could be purchased from standard commercial sources. Compounds **1–3** were all prepared using commercial mexiletine which is a racemate and hence exist as diastereomeric mixtures, although these are not resolved in the compounds’ ^1^H NMR spectra. Given the racemic nature of the target drug, mexiletine, no attempt was made to prepare enantiomerically or diastereomerically pure gelators. Full experimental details and characterization data for all three gelators can be found in the Supporting Information.

## Gel Characterization

The gelation behavior of compounds **1**, **2**, and **3** was tested in 46 solvents, spanning a wide range of chemical functionality. A 2% w/v solution of the gelator was heated to its boiling point in a sealed vial using a heat gun. The solution was placed in an insulating wooden block at room temperature and monitored for 24 h. Gelation was identified by the inversion test.^23^ If the material supported its own weight and did not flow when the vial was inverted, the material was classed as a gel (although this method can give false positives in the case of viscous liquids). The results of gel screening are shown in Table S1. Compound **1** proved to be the most versatile gelator, gelling 35 out of the 46 solvents tested, whereas compound **2** gelled 13 solvents and compound **3** gelled 8. This pattern reflects previous studies, in which compounds based on linker **1** often prove to be the most effective gelators.^28,39,42,49^ Of the 46 solvents used for gel testing, 20 were included in the solution-phase polymorph screen of mexiletine described in previous work.^36^ Including partial gels, compound **1** gelled 15 of these solvents, compound **2** gelled 3, and compound **3** did not gel any. These 20 solvents were used for most of the gel-phase crystallization experiments to facilitate comparison with the results of the solution-phase crystallization data as a control.

**Figure 4:**
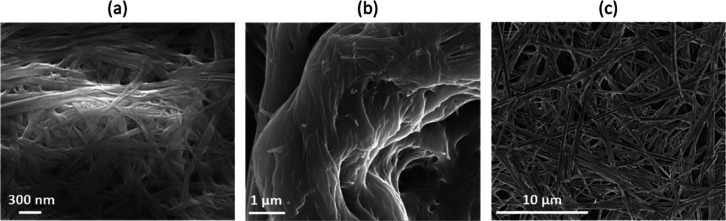
SEM micrographs of dried xerogels prepared from (a) 1% (w/v) gel of compound **1** in nitrobenzene, (b) 2% (w/v) gel of compound **2** in nitrobenzene, and (c) 2% (w/v) gel of compound **3** in nitrobenzene. Samples were coated in 7 nm gold–palladium.

At a concentration of 2% w/v, gels of compound **1** were either opaque or contained visible particles of the undissolved gelator. Transparent gels of compound **1** could be achieved by reducing the concentration to 1% w/v, although solid gelator particles were unavoidable in gels of apolar or low boiling point solvents. Compounds **2** and **3** were more soluble and these gels were transparent. Scanning electron microscopy (SEM) images of the dried xerogels prepared from compounds **1**, **2**, and **3** in nitrobenzene (chosen for the robustness of the gels and high boiling point of the solvent) all showed a fibrillar network characteristic of a supramolecular gel (Figure 4).^60^

Oscillatory rheology was used to probe the mechanical properties of representative gels. A 1% w/v gel of compound **1**, and 2% w/v gels of compounds **2** and **3** in 1,2,4-trichlorobenzene (TCB) were characterized using this technique. This solvent was chosen because its high boiling point and low vapor pressure produced uniform gels that did not dry out during the measurement (Figure S6). A lower concentration was used for compound **1** because this gelator has a lower solubility, and gels at 2% w/v concentration contained undissolved solid that may alter their rheological properties.

**Figure 5:**
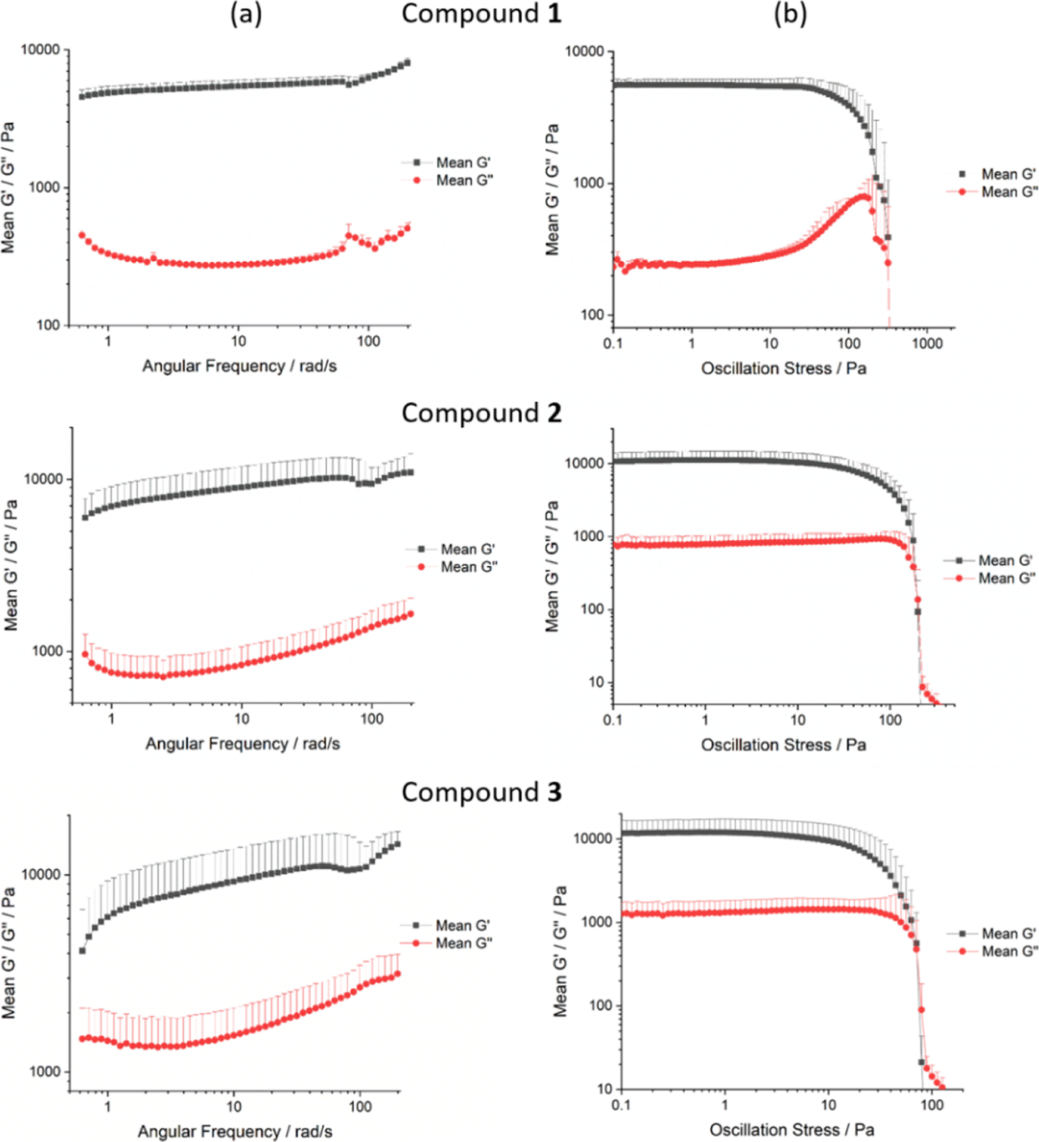
Oscillatory (a) frequency and (b) stress sweeps for a 1% w/v gel of compound **1** in TCB, and 2% w/v gels of compounds **2** and **3** in TCB. Error bars indicate the standard deviation from repeated measurements. For clarity, only the positive error bars are displayed.

The gel phase can be identified by a storage modulus approximately 1 order of magnitude greater than the loss modulus, which does not vary with frequency.^61^ This linear region was observed in the frequency sweep data for all gels, between 0.6 and 210 rad/s (Figure 5a), and confirms that these materials all display the elastic behavior characteristic of a gel. The yield stress of a gel, which is used to quantify its strength, can be identified from stress sweep data as the oscillation stress at which the storage and loss moduli are equal (Figure 5b). The bis(urea) gels of compounds **1** and **2** were significantly stronger than the trisamide gel of compound **3**, possibly due to the greater number of hydrogen bond donors although trisamide gels can be quite robust.^62^ Despite the lower concentration, compound **1** produced the strongest gel, with a yield stress of *ca.* 320 Pa. The gel of compound **2** had a comparable yield stress of *ca*. 200 Pa, whereas compound **3** produced a much weaker gel, with a yield stress of *ca*. 70 Pa. This trend mirrors previous reports in which bis(urea) gelators containing the linking group in compound **1** are stronger than those based on the linking group in compound **2**.^28,39,42,49^ A weak strain overshoot was observed in gels of compound **1**, where the loss modulus, *G*″, increases just before the yield stress is reached. This behavior is indicative of a second mode of aggregation, in which components of the gel fiber align in the direction of the applied shear, forming a weak structure that is capable of resisting deformation for a short time, before it yields and the gel begins to flow.^63^ Weak strain overshoot is common in systems containing hard particles.^64,65^ The low solubility of compound **1** may have led to precipitation within the gel, which could have contributed to this behavior.

## Solution-Phase Polymorph Screening

Compound **3** did not gel any of the solvents that were included in the previous solution-phase polymorph screen. Therefore, further solution-phase crystallizations of mexiletine hydrochloride were carried out in the six solvents that were gelled by all three gelators, so that these results could be compared to gel-phase crystallizations using all three gelators. Solution-phase crystallization was carried out by slowly cooling a supersaturated solution of mexiletine, formed by dissolving 20 mg of mexiletine powder in the minimum possible solvent and heating it to boiling in a sealed glass vial. The mixture was allowed to cool to room temperature in an insulating wooden block and monitored for crystallization over time. Powder X-ray diffraction (PXRD) was used to assess the solid form of the resulting crystals, as summarized in Table 1.

**Table 1:** Solid Form of Mexiletine Hydrochloride Samples Crystallized by Slow Cooling from a Supersaturated Solution in 1,2-Dibromoethane, Chlorobenzene, 1,2-Dichlorobezene, 1,3-Dichlorobenzene, TCB, and Nitrobenzene

solvent	polymorph
chlorobenzene (CB)	type A solvate
1,2-dichlorobenzene (12DCB)	type A solvate
1,3-dichlorobenzene (13DCB)	type A solvate
1,2-dibromoethane	type B solvate
TCB	type C solvate
nitrobenzene (NB)	type D solvate

**Figure 6:**
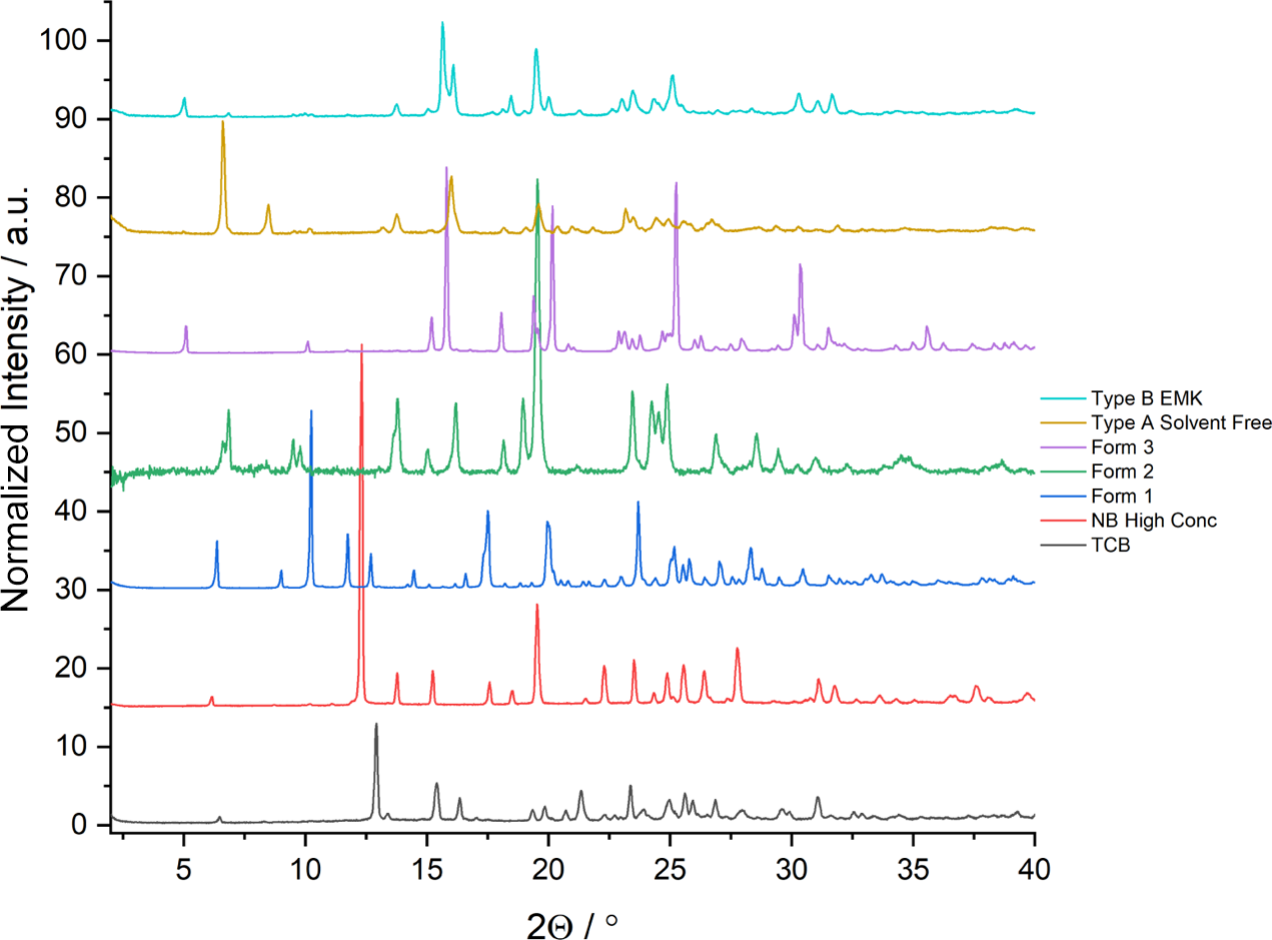
PXRD patterns of type C mexiletine hydrochloride solvate crystallized by slow cooling from TCB, compared to the type D nitrobenzene solvate, and the five previously known forms (1–3 and types A and B). For clarity, one representative example of type A and type B solvates is shown.

Four of the solution-phase crystallizations led to known forms characterized previously by solution-phase polymorph screening.^36^ Crystallization from the chlorinated solvents chlorobenzene, 1,2-dichlorobenzene, and 1,3-dichlorobenzene yielded type A solvates with PXRD patterns closely related to the type A diethyl ether solvate crystallized by vapor diffusion of diethyl ether into a saturated solution of mexiletine in dimethylformamide (DMF) (Figure S7).^36^ Crystallization from 1,2-dibromoethane produced a type B solvate, with a PXRD pattern that closely resembles the type B solvate crystallized by slow cooling from ethyl methyl ketone (EMK) (Figure S8).^36^ Two new solvated polymorphs of mexiletine were reproducibly crystallized from TCB and nitrobenzene (three repeats). Their PXRD patterns are not related to any of the three known pure forms nor the known type A and B solvates.^36^ Therefore, they will be referred to as the type C TCB solvate and the type D nitrobenzene solvate. In addition to the five previously known forms, the discovery of these new solvates means that mexiletine has seven known solid form types. The PXRD pattern of the type C polymorph contains, for example, unique peaks at 12.9, 15.4, and 16.4° 2θ that are not observed in any of the previously known forms (Figure 6).

A single-crystal structure of type C solvate was determined using a crystal grown by slow cooling of a supersaturated solution of mexiletine in TCB. Full crystallographic information for this structure is given in Table S2. The type C structure is a 4:1 mexiletine/TCB tetarto-solvate,^66^ in which the solvent molecules are situated inside channels that run along the *a*-axis of the mexiletine host framework. The structure is a racemate, with the asymmetric unit containing two identical pairs of mexiletine molecules and one TCB molecule. The two symmetry-independent mexiletine molecules both adopt a gauche conformation, with O–C–C–N torsion angles of 62.2 and 58.1°, which are in line with other structures containing the R–O–CH_2_–CHR–NH_3_^+^ fragment in the Cambridge Structural Database. Each ammonium cation hydrogen bonds with three chloride counterions, forming a hydrogen-bonded polymer along the crystallographic *a*-axis. When viewed along this axis, the molecules are arranged in a square formation, versions of which are observed in all forms of mexiletine other than form 1. In this case, the four molecules making up the square motif are related by inversion, as shown in Figure S9a. Although the solvent molecules in this structure are disordered, it was possible to model them without using a solvent mask and they are clearly visible within the channels (Figure S9b). In contrast to the type A solvates, which are typically disordered, the ordering is likely to arise from the limited number of positions that the large trichlorobenzene molecule can occupy within the small channel.

**Figure 7:**
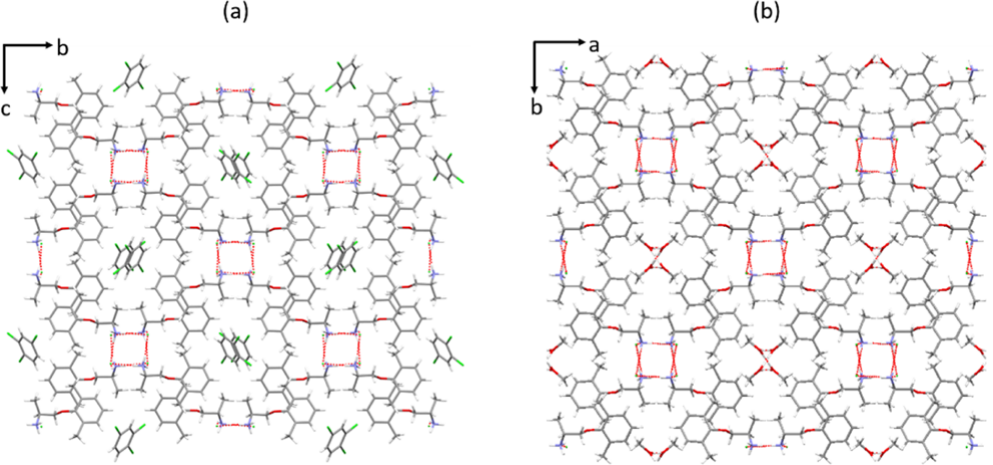
Packing arrangements in the type C TCB solvate of mexiletine, compared to the type A methanol solvate.

Although the type C solvate has a different symmetry, the packing arrangement, hydrogen bonding motifs, and unit cell dimensions are closely related to type A solvates. When compared to the type A methanol solvate, which is the only member of that family in which the solvent molecules are clearly resolved in the crystal structure, several similarities are visible. Viewed down the channels, the packing arrangement of molecules within the mexiletine framework of the two solvates is nearly identical. Both structures consist of offset layers that alternate every half unit cell, so the channels line up every other layer (Figure 7). The molecules are arranged very differently down the other two axes, although the hydrogen bonding motifs between molecules are closely related. There are slight differences in the unit cell dimensions of these two forms, which reflect changes in the channel dimensions to accommodate different solvents (Table S3).

When stored for 24 h under ambient conditions, the type C trichlorobenzene solvate transformed into a type A solvate, producing a PXRD pattern that closely matched the type A diethyl ether solvate crystallized by vapor diffusion (Figure S10). The type A solvates are metastable with respect to form 1, so this unusual result suggests that the trichlorobenzene solvate may be very close in energy to the type A solvates. As the two forms are structurally similar, only a small degree of molecular rearrangement is required during the transformation, which is reflected in the high crystallinity of the sample after storage.

The polymorphic outcome of the crystallizations from nitrobenzene proved to depend on the concentration of mexiletine. Low concentrations produced form 1 (the thermodynamic form under ambient conditions), whereas higher concentrations led to the type D nitrobenzene solvate (Figure S11). The PXRD pattern of this form lacks several key peaks from each of the known forms and contains a readily recognized, unique peak at 12.3° (Figure 6). Form 1 has a very characteristic IR spectrum,^36,67^ so IR spectroscopy was used to identify the concentration that favors the type D polymorph over form 1. Seven solutions were prepared at varying concentrations, according to Table S4. When 20 mg of mexiletine was dissolved in 0.15 mL of nitrobenzene or less (>13.3% w/v), the new form D was produced, whereas solvent volumes of 0.2 mL and above (<10% w/v) led to form 1.

The type D solvate can only be crystallized at high degrees of supersaturation, which suggests that it is metastable, and accordingly, it transforms into a mixture with form 1 when stored for 24 h under ambient conditions (Figure S12).

The type C and type D solvates were further characterized by IR spectroscopy. Both forms have unique spectra, different from each other and the known forms (Figure S13). Both spectra also contained peaks assigned to the included solvent, which confirms that they are solvates. In the type C TCB solvate, the solvent peaks occur at 1457, 866, 815, and 678 cm^–1^. Whereas, in the type D nitrobenzene solvate, these peaks occur at 1527, 1350, 1317, 852, 843, and 682 cm^–1^. From this data, it is not possible to know how the solvent molecules are incorporated into the type D crystal structure. However, given that all previous solvated forms are channel solvates, it is likely that this form has a similar structure. Similarly, there are likely to be more possible type C solvates, incorporating different solvents into the channels.

It was not possible to characterize the type C and type D solvates by differential scanning calorimetry and thermogravimetric analysis, because the extremely low vapor pressure of the solvents they were crystallized from caused significant amounts of solvent to adsorb onto the surface of the powders, meaning that the thermograms mostly contained features assigned to the unbound solvent. The powders could be dried in a desiccator or a low-temperature oven, but by the time the solvent had evaporated, the samples had changed form.

## Gel-Phase Crystallization

**Figure 8:**
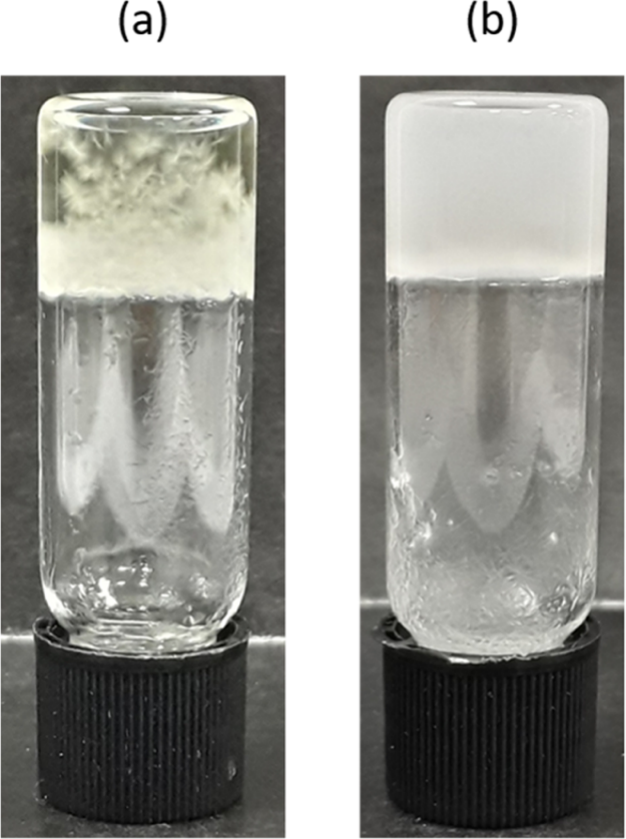
Gel-phase crystallization of mexiletine in (a) compound **2**, at a concentration of 2% w/v gelator 5% w/v drug in nitrobenzene and (b) compound **1**, at a concentration of 1% gelator 5% w/v drug in 1,2-dichlorobenzene. In the results given in Tables S5–S7, samples like image (a) are described as gel + crystals, whereas samples like image (b) are described as gel + precipitate.

Gel-phase crystallization of mexiletine was carried out using gels of all three gelators, in solvents that were included in the solution-phase polymorph screens. The concentrations of the drug and gelator were optimized to ensure that where possible, gelation occurred before crystallization, so the gel network could interact with the crystallizing drug molecules. Compound **1** is sparingly soluble in most solvents, so a low concentration of gelator was used in these experiments. The drug and gelator were dissolved in 0.5 mL of the required solvent by heating the mixture to the boiling point of the solvent in a sealed glass vial. The vials were placed in an insulating wooden block and monitored for gelation and crystallization (Figure 8). After 24 h, the vials were emptied onto filter paper, left to dry in air, and the resulting powder was characterized by PXRD. PXRD patterns of the gel-grown samples were compared to the solution-phase materials crystallized from the same solvent at the same concentration, to establish whether any change in polymorphism had occurred due to the presence of the gel network.

All gel-phase crystallizations using compound **3** resulted in the same solid forms as obtained from solution (Table S5). Crystallization within gels of 1,2-dichlorobenzene, 1,3-dichlorobenzene, and chlorobenzene led to type A solvates, gels of 1,2-dibromoethane crystallized a type B solvate, and gels of TCB and nitrobenzene produced type C and type D solvates, respectively. Gels of compound **3** are an order of magnitude weaker than both the bis(urea) gels, and perhaps contain fewer gel fibers. The gel network may therefore have been insufficient to encourage the growth of new polymorphs, leading to the same crystallization behavior as in solution.

Mexiletine crystallized within 13 of the 15 gel-phase crystallization experiments using compound **1** (Table S6). Due to its high solubility in polar solvents, mexiletine did not crystallize in any gel containing ethanol or methanol and the resulting PXRD patterns matched the gelator alone. Most gel-phase crystallization experiments using compound **1** yielded the same solid form as obtained from solution. Form 1 was crystallized from gels of nitromethane, 1-propanol, 2-propanol, 1-butanol, 2-butanol, and amyl alcohol. Type A solvates crystallized from gels in all chlorinated solvents: DCM, 1,2-dichlorobenzene, 1,3-dichlorobenzene, and chlorobenzene, and type B solvates crystallized from gels in THF and dioxane. Similarly, gels of TCB and nitrobenzene produced type C and type D solvates, respectively. Incorporation of mexiletine inhibited the gel formation of compound **1** in acetonitrile and accordingly, the result of these crystallizations was also the same as in solution. At concentrations of 1% w/v gelator and 2% w/v drug, mexiletine crystallized as form 1, as observed in slow cooling crystallizations from pure acetonitrile. At a higher supersaturation, using 2% w/v of gelator and 5% w/v of drug, mexiletine crystallized as the metastable form 3. This result mirrors solution-phase behavior in which a mixture of forms 1 and 3 can be crystallized by fast cooling from pure acetonitrile.

**Figure 9:**
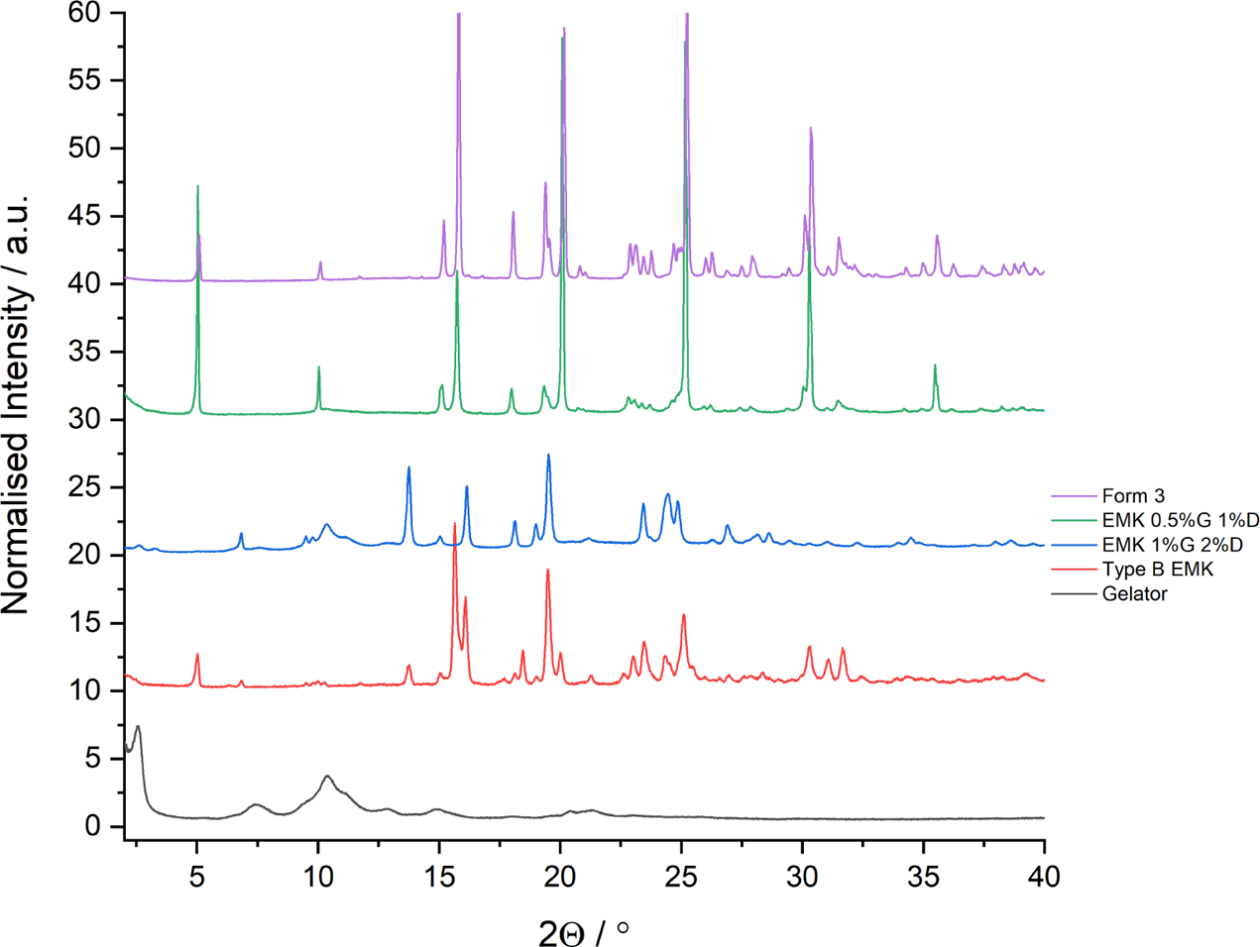
PXRD patterns of the two solid forms of mexiletine crystallized within two EMK gels of compound **1**, compared to form 3, the type B EMK solvate, and compound **1**.

In contrast, gel-phase crystallizations using compound **1** in EMK, DMF, and DMSO produced different solid forms to those obtained from solution. A type B solvate crystallized from EMK solution, and the same form was observed from a gel containing 1% w/v gelator and 2% w/v drug. However, when the concentrations were reduced to 0.5% w/v gelator and 1% w/v drug, form 3 was produced (Figure 9). Form 3 is a metastable polymorph, very close in energy to form 1,^36^ and pure samples have only been crystallized previously from solution in acetone. The crystallization of pure form 3 within this drug-mimetic gel highlights its ability to stabilize and selectively nucleate a metastable solid form.

Finally, a crystalline solid form is produced from gels of compound **1** in DMF and DMSO, whereas mexiletine does not crystallize from solution in either of these solvents. The PXRD patterns of the gel-crystallized samples all contain gelator peaks, showing that only a small amount of the drug has crystallized. The crystallinity of the samples increases with drug concentration, and at 1% w/v gelator and 10% w/v drug, some clear mexiletine peaks are observed at 4.9, 6.4, 19.5, 19.9, 24.4, 25.0, 29.3, and 30.0° (Figure S14). These peaks match most closely with the type A diethyl ether solvate, which is crystallized by vapor diffusion of diethyl ether into DMF. Although the low crystallinity of their PXRD patterns means that it is not possible to assign the polymorphism of these gel-grown crystals unequivocally, they are likely to be type A solvates because that form can also be crystallized from DMF by vapor diffusion. A similar behavior was observed when sulfapyridine was crystallized from a nanocellulose organogel in DMSO. Crystallization was not observed from solution under the same conditions, even though the solution was highly supersaturated, and the gel network was thought to be acting as a kinetic nucleation promoter.^33^ It is therefore likely that in this case, the gel fibers are acting as nucleation sites to enable the crystallization of a type A solvate from an unusual solvent.

The greatest differences between gel and solution phase polymorphism were observed when mexiletine was crystallized using compound **2**. The polymorphic outcome of these crystallizations was dependent on the concentration of mexiletine, and in many cases, gelation was switched off in experiments that led to a change in the solid form. This behavior suggests that there were significant interactions between the drug and gelator molecules that hindered the self-assembly of gel fibers.^35^ It is likely that the strong interactions between the drug and gelator molecules play a key role in the nucleation of unusual solid forms. Due to the inconsistent gelation behavior of this system, experiments were repeated multiple times, so that a reliable trend could be established (Table S7).

**Figure 10:**
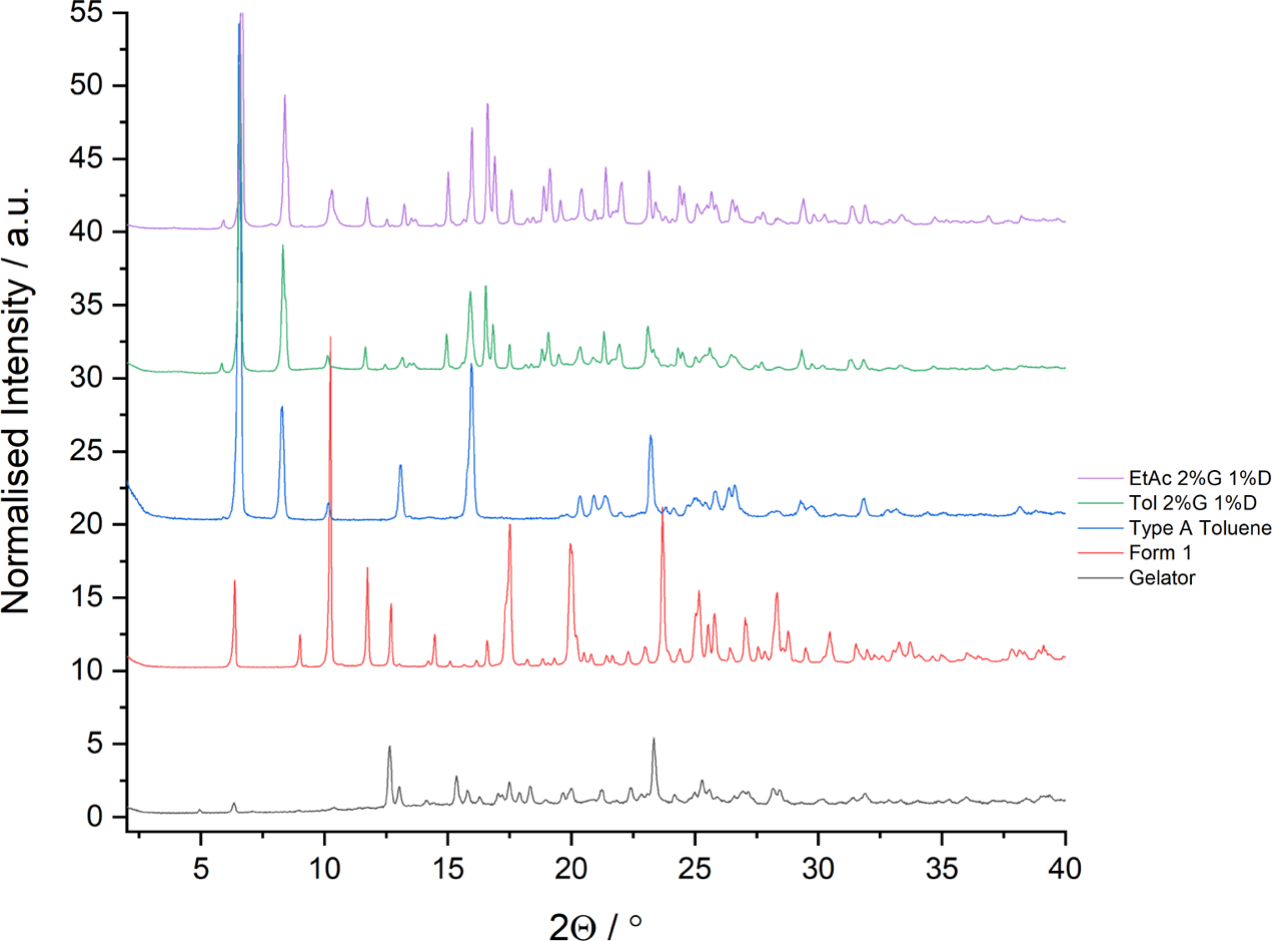
PXRD patterns of mexiletine crystallized from toluene and ethyl acetate solutions, at concentrations of 2% w/v gelator and 1% w/v drug, compared to form 1, the type A toluene solvate, and the gelator.

In several cases, the same solid form crystallized from gels as from solution. Form 1 crystallized from gels of compound **2** in nitromethane, and type A solvates crystallized from gels in 1,2-dichlorobenzene, 1,3-dichlorobenzene, and chlorobenzene. Similarly, type C and type D solvates crystallized from gels of TCB and nitrobenzene, respectively. The majority of crystallizations from toluene also produced the same form as in solution: a type A solvate. However, in one crystallization with a low drug concentration of 1% w/v, a new solid form was produced. The PXRD pattern of this form contains the key peaks characteristic of a type A solvate and many extra peaks between 12 and 27° that are not present in the pattern of the toluene solvate crystallized from solution (Figure 10). The extra peaks in the PXRD pattern of the gel form suggest that the contents of the channels differ from the solution form. This new type A solvate also crystallized from ethyl acetate at concentrations of 2% w/v gelator and 1% w/v mexiletine (Figure 10). This result is particularly unusual because mexiletine crystallizes as form 1 from ethyl acetate solution. In both of these cases, gelation was switched off by interactions between mexiletine and the gelator.^35^

Two other ethyl acetate crystallizations, with concentrations of 2% w/v gelator and 2 or 5% w/v drug, also led to a type A solid form, although in these cases, the PXRD pattern matched the type A solvent-free structure (Figure S15). In this form, the channels may be empty or could be filled with the highly disordered solvent that does not diffract X-rays. It is clear that compound **2** has a profound effect on the nucleation of the type A solvates, and the crystallization of the solvent within the channels.

**Figure 11:**
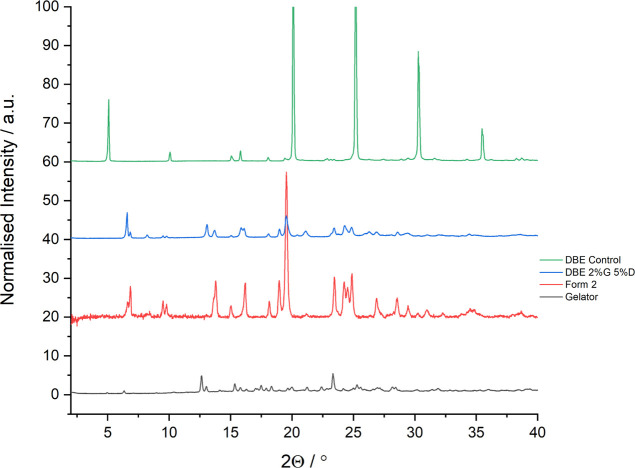
PXRD pattern of the mexiletine solid form crystallized from a gel of compound **2** in 1,2-dibromoethane, compared to form 2, the type B form crystallized from 1,2-dibromoethane solution (DBE control), and the solid gelator.

Finally, the non-solvated high temperature stable form 2 crystallized from a gel of compound **2** in 1,2-dibromoethane (Figure 11). Form 2 is extremely unstable at room temperature and previously, has only been crystallized by sublimation or by heating another form above its transition temperature. Crystallization within this gel is therefore the only known method to access form 2 at room temperature. The sample gels before crystallization and gelation are not disrupted by the presence of the solute, which suggests that the nucleation processes of the drug and gelator occur on different timescales, likely driven by the high solubility of mexiletine in 1,2-dibromoethane. As a result, the gel network forms before the crystals began to grow, potentially facilitating epitaxial overgrowth of crystals upon the gel fibers and stabilizing this extremely high energy solid form.

## Conclusions

In conclusion, this work demonstrates the versatile gelation behavior of three mexiletine-mimetic supramolecular gelators. Significant changes in polymorphism were observed when the API mexiletine HCl was crystallized within the two bis(urea) gels. Gels of compound **1** in DMF and DMSO facilitated the crystallization of a type A solvate, in solvents from which mexiletine does not crystallize in solution. Similarly, in an EMK gel of compound **1**, mexiletine crystallized as form 3, which is metastable and often crystallizes concomitantly with form 1. This gel is only the second known route to access a pure sample of form 3, which shows that the gel network can selectively nucleate a metastable solid form. Similarly, the metastable form 2 was crystallized from a 1,2-dibromoethane gel of compound **2**. Form 2 is the high temperature stable polymorph of mexiletine and is significantly higher in energy than all the other forms. Crystallization within this gel is the only known route to access this form at room temperature, which demonstrates the powerful stabilizing effect of this gel network. Compound **2** also enabled the crystallization of unusual type A solvates. Crystallization from ethyl acetate solutions of compound **2** at drug concentrations of 2 and 5% w/v presented a new route to a known type A structure. Whereas, a new type A solvate was crystallized from the same mixture at a lower drug concentration of 1% w/v. This novel type A solvate can also be accessed from solutions of compound **2** in toluene at 1% w/v concentration of mexiletine. In these experiments, the mixture did not form a gel, which suggests that interactions between the drug and the gelator inhibited the self-assembly of gel fibers. It is likely that these interactions are responsible for the changes in polymorphism observed in these experiments. These results show the versatile ability of drug-mimetic supramolecular gels to achieve solid-form modification of an API. Finally, two additional solvated polymorphs of mexiletine were crystallized from solutions in TCB and nitrobenzene, which further highlights the prolific solvate-forming behavior of this compound. A crystal structure of the type C trichlorobenzene solvate showed that it is another channel solvate, which suggests that there may be more modifications of this polymorph to be found.

## Supporting Information Available

Materials and instrumentation used in this study; full synthesis and characterization data for the three gelators reported in this work; gel screening results, PXRD patterns, IR spectra, and full crystallographic information for the type C TCB solvate; and results of all gel-phase crystallization experiments (PDF)
