# Supplementary material for: Designer Gelators for the Crystallization of a Salt Active Pharmaceutical Ingredient—Mexiletine Hydrochloride
Source: Cryst Growth Des. 2022 Oct 12;22(11):6775–85. doi: 10.1021/acs.cgd.2c00925 (PMC9635620; doi:10.1021/acs.cgd.2c00925)
Supplement: Supplementary file 1 — cg2c00925_si_001.pdf [file cg2c00925_si_001.pdf]

Designer Gelators for the Crystallisation of a Salt Active Pharmaceutical Ingredient - Mexiletine Hydrochloride

Jessica L. Andrews, Stuart R. Kennedy[†], Dmitry S. Yufit, Jonathan W. Steed*.

Department of Chemistry, Durham University, Durham, DH1 3LE, UK.

Electronic Supplementary Information

Experimental Details

Materials:

All solvents and starting materials were purchased from standard commercial sources and used without further purification.

Instrumentation:

Powder X-Ray Diffraction, PXRD

Powder X-ray diffraction, PXRD, was performed using a Bruker D8 X-ray diffractometer in Bragg-Brentano geometry. Samples were mounted on a silicon single-crystal wafer and analysed using Cu-Kα radiation at a wavelength of 1.5406 Å. X-rays were produced using an operating voltage of 40 kV and current of 40 mA. Samples were scanned over an angle range of 2-40° 2θ, with a step size of 0.02 ° and a scan rate between 0.5-1.5 s/step.

Single-Crystal X-Ray Diffraction

Single crystal data for **Type C** crystal were collected at 100.0(2)K at I-19 beamline (Dectris Pilatus 2M pixel-array photon-counting detector, undulator, graphite monochromator, λ = 0.6889 Å) at the Diamond Light Source, Oxfordshire and processed using Bruker APEX-III software. The structure was solved by direct method and refined by full-matrix least squares on F^2 for all data using Olex2[1] and SHELXTL[2] software. All non-disordered non-hydrogen atoms

were refined in anisotropic approximation, hydrogen atoms were placed in the calculated positions and refined in riding mode. Carbon atoms of disordered trichlorobenzene solvent molecule were refined isotropically with fixed occupancy of 0.5. The Cl-C bond lengths in the solvent molecules were restrained to be the same with esd of 0.005Å, the carbon atoms there were refined as a regular hexagon. Crystal data and parameters of refinement are listed in Table S2. Crystallographic data for the structure have been deposited with the Cambridge Crystallographic Data Centre as supplementary publication CCDC-2191015.

Infra-red Spectroscopy

Fourier transform infra-red spectroscopy (FTIR) was carried out using either a Perkin Elmer Spectrum 100 spectrometer, fitted with a diamond universal Attenuated Total Reflectance (ATR) accessory. Four scans were collected for each sample at a resolution of 2 cm^{-1} over a wavenumber region of 4000 cm^{-1} to 600 cm^{-1}.

Thermogravimetric Analysis, TGA

Thermogravimetric analysis, TGA, was carried out using a TA Instruments Q 500 TGA analyser. Between 1 and 5 mg of sample was weighed into platinum pans and dry nitrogen was used as the purge gas (flow rate: 60 mL min^{-1}).

Differential Scanning Calorimetry, DSC

Differential scanning calorimetry, DSC, was performed using a Perkin Elmer 8500 calorimeter, calibrated using an indium standard (melting point onset = 156.6 °C, heat of fusion = 28.57 J g^{-1}). Between 1 and 3 mg of sample was weighed accurately (±0.01 mg) using a Sartorius microbalance into sealed aluminium pans and dry nitrogen was used as the purge gas (flow rate: 50 mL min^{-1}).

Synthesis and Characterisation of Compounds 1,2 and 3:

Synthesis and characterisation of bis(3,5-diethyl-4-isocyanatophenyl)methane

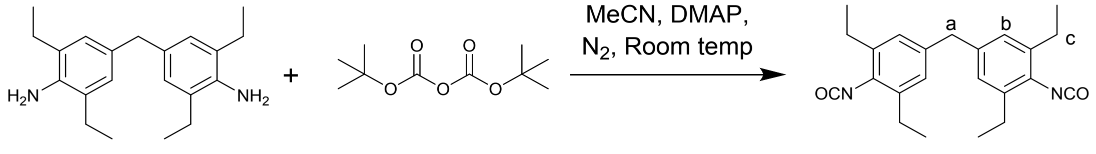

Bis(3,5-diethyl-4-isocyanatophenyl)methane was synthesised according to the literature method.[3] A solution of di-tert-butyl dicarbonate (6.00 g, 34.4 mmol) in dry acetonitrile (20 mL) was slowly added to a solution of 4-DMAP (0.33 g, 2.70 mmol) in dry acetonitrile under a flow of nitrogen. A solution of 4,4'-methylenebis(2,6-diethylaniline) (4.00 g, 12.9 mmol) in dry acetonitrile (20 mL) was slowly added to the previous solution and the resulting mixture was stirred for 2 hours at room temperature under nitrogen. Concentrated H_2SO_4 (2 mL) was slowly added to acetonitrile (3 mL), this solution was added to the reaction mixture and

stirred for 5 minutes. The reaction was quenched with water (65 mL) and the solution was extracted with hexane (4 × 100 mL). The combined hexane extracts were dried over $MgSO_4$, filtered and the solvent was removed under vacuum. The crude product was dissolved in DCM (20 mL) and the solution was filtered to remove any solid impurities. The solvent was removed under vacuum, to yield bis(3,5-diethyl-4-isocyanatophenyl)methane as a white solid (1.59 g, 4.39 mmol, 34 %). This compound was used without further purification, for the synthesis of compound **1**. The characterisation data for this compound were consistent with previous literature.[3]

^{1}H NMR ($CDCl_3$, 400 MHz): δ 6.91 (s, 4H, b), 3.90 (s, 2H, a), 2.70 (q, J = 7.6 Hz, 8H, c), 1.26 (t, J = 7.6 Hz, 12H, d).

$^{13}C\{^{1}H\}$ NMR ($CDCl_3$, 101 MHz): δ 138.93 (C=O), 138.53 (Ar$\underline{C}$), 127.99 (Ar$\underline{C}$), 126.97 (Ar$\underline{C}$), 123.81 (Ar$\underline{C}$), 41.15 (Ar-$\underline{C}H_2$-Ar), 25.73 (Ar-$\underline{C}H_2$-CH_3), 14.27 (Ar-CH_2-$\underline{C}H_3$).

m/z ESI-MS (MeCN): retention time 4.05 min 363.4 $[M+H]^+$. The $[M+H]^+$ peak is very low intensity and there are much larger peaks corresponding to the fragment molecule in which one isocyanate group has broken down to an amine: 337.4 $[M+H]^+$.

Synthesis and characterisation of Compound 1

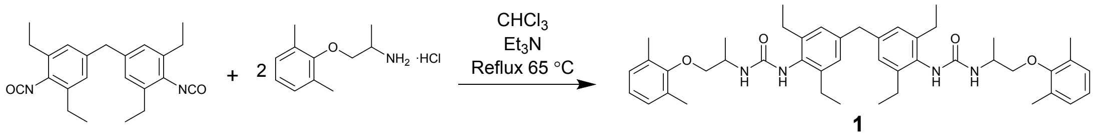

1

Racemic mexiletine hydrochloride (1.45 g, 6.7 mmol) was suspended in chloroform (100 mL) and dissolved upon the addition of excess triethylamine (1 mL). Bis(3,5-diethyl-4-isocyanatophenyl)methane (1.00 g, 3.06 mmol) was added, and the solution was heated to reflux and stirred for 24 hours. The crude product was collected by filtration, suspended in water (100 mL) and sonicated for 10 minutes to remove water soluble impurities. The precipitate was collected by filtration, washed with water (100 mL) and chloroform (50 mL), and dried under vacuum to yield compound **1** as a white solid (1.90 g, 2.63 mmol, 86 %).

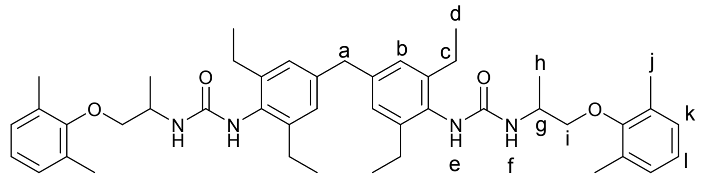

Compound **1** had limited solubility in all common NMR solvents. In cases where sufficient compound dissolved to produce a high-resolution ^{1}H NMR spectrum, the sample gelled (**Figure S1**

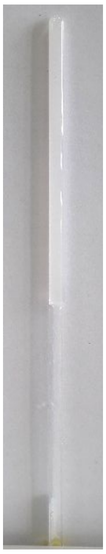

Figure S1) and as a result, some multiplicity information was lost due to significant peak broadening. For these reasons, it was not possible to obtain a solution-state ^{13}C NMR spectrum of this molecule, and CP MAS SS NMR was used instead.

^{1}H NMR ($CDCl_3$, 400 MHz): 7.05-6.87 (m, 10H, b, k, l), 5.65 (s br, 2H, e), 4.78 (apparent t, J = 7.6 Hz, 2H, f)†, 4.28-4.17 (m, 2H, g/H_X), 3.92 (s, 2H, a), 3.72-3.60 (m, 4H, i/$H_{A,B}$), 2.61 (s br, 8H,

c), 2.03 (apparent d, J = 4.3 Hz, 12H, j)†, 1.36 (apparent dd, J = 6.7, 5.1 Hz, 6H, h)†, 1.16 (t, J = 7.6 Hz, 12H, d).

† Compound 1 was synthesised using a racemic starting material and the product is therefore a mixture of meso and rac diastereoisomers. As a result, peaks in the ^{1}H NMR spectrum corresponding to environments ‘f’, ‘j’ and ‘h’ have a higher apparent multiplicity due to the overlapping of signals from each diastereoisomer.

Due to the lower resolution of solid-state NMR, many peaks in the ^{13}C spectrum overlap, and the spectrum is also complicated by the presence of multiple diastereoisomers. Tentative peak assignments are given, based on the spectra of other mexiletine-terminated gelators.

^{13}C NMR (CP-MAS SS NMR, 101 MHz): 158.00 ($\underline{C}$=O), 154.58 (Ar$\underline{C}$), 141.05 (Ar$\underline{C}$), 132.58 (Ar$\underline{C}$), 130.81 (Ar$\underline{C}$), 129.78 (Ar$\underline{C}$), 127.81 (Ar$\underline{C}$), 125.89 (Ar$\underline{C}$), 123.91 (Ar$\underline{C}$), 76.10 (Ar-O-$\underline{C}H_2$), 45.73 (NH-$\underline{C}$H-CH_3), 42.74 (Ar-$\underline{C}H_2$-Ar), 24.30 (AR-$\underline{C}H_2$-CH_3), 23.20 (O-Ar-$\underline{C}H_3$), 15.88 (NH-CH-$\underline{C}H_3$), 13.47 (Ar-CH_2-$\underline{C}H_3$).

m/z ESI-MS (MeCN): retention time 3.91 min, 721.7 $[M+H]^+$.

Elemental Analysis: Calc. (%): C 74.96, H 8.39, N 7.77; Found (%): C 74.74, H 8.32, N 7.69.

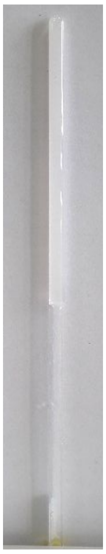

Figure S1. A gelled NMR sample of compound **1** in $CDCl_3$.

Synthesis and characterisation of Compound 2

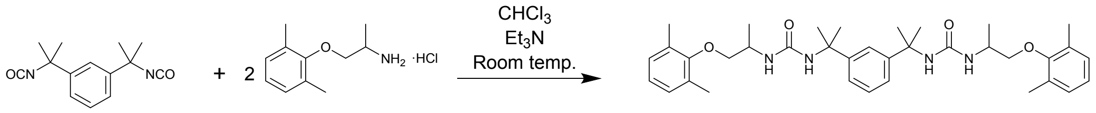

Racemic mexiletine hydrochloride (1.42 g, 6.6 mmol) was suspended in chloroform (80 mL) and dissolved upon the addition of excess triethylamine (1 mL). 1,3-Bis(1-isocyanato-1-methylethyl)benzene (0.69 mL, 3 mmol) was added and the solution was stirred at room temperature for 24 hours. The solvent was removed under vacuum and the crude product was sonicated for 10 minutes in water (100 mL) and then acetonitrile (50 mL), to yield compound **2** as a white solid (1.31 g, 2.18 mmol, 73 %).

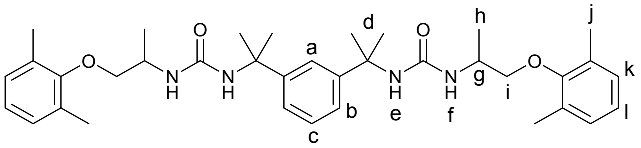

^{1}H NMR (DMSO-d6, 599 MHz): δ 7.33 – 7.29 (m, 1H, c), 7.17 – 7.10 (m, 3H, a, b), 6.97 (d, J = 7.5 Hz, 4H, k), 6.87 (t, J = 7.5 Hz, 2H, l), 6.28 (s, 2H, e), 5.94 (d, J = 8.2 Hz, 2H, f), 3.86 – 3.78 (m, 2H, g/H_X)†, 3.58 (ABq, J_{AB} = -9.5 Hz, J_{AX} = 1.61 Hz, 2H, i/H_A)†, 3.56 (ABq, J_{AB} = -9.5, J_{BX} = 0.2 Hz, 2H, i/H_B)†, 2.19 (s, 12H, j), 1.48 (apparent t, J = 5.6 Hz, 12H, d)*, 1.19 (d, J = 6.7 Hz, 6H, h).

† See Figure S2 for further details of the assignment of these peaks.

*See Figure S3 for further details of the assignment of these peaks.

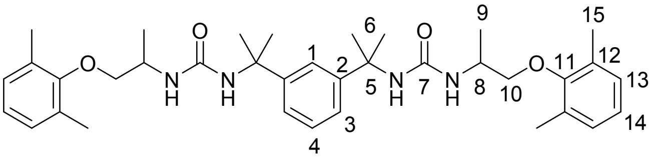

13C {1H} NMR (DMSO-d6, 151 MHz,) δ 157.00 (C7), 155.52 (C12), 148.86 (C2), 130.80 (C11), 129.17 (C13), 127.69 (C1), 124.10 (C14), 122.77 (C3), 121.71 (C4), 75.15 (C10), 54.72 (C5), 45.34 (C8), 30.67 (C6)*, 30.61 (C6)*, 30.52 (C6)*, 30.46 (C6)*, 18.52 (C9), 16.32 (C15).

*See Figure S3 for further details of the assignment of these peaks.

m/z ESI-MS (MeCN): retention time 3.40 min, 603.9 [M+H]$^{+}$, 1205.8 [2M+H]$^{+}$.

Elemental Analysis: Calc. (%): C 71.73, H 8.36, N 9.29; Found (%): C 71.32, H 8.24, N 9.29.

Protons ‘i’ and ‘g’ make up an ABX system as shown in Figure S2. The signals corresponding to H_A and H_B (environment i) could be assigned as two AB quartets however, the difference in chemical shift between the two signals is very small, so the central peak overlaps, and the signal contains 7 lines instead of 8. The pure shift signal for protons ‘i’ is an apparent triplet, in which the central peak is an artefact that indicates strong coupling between the two

geminal protons. The proton signal corresponding to H_X (environment g) is further complicated by coupling to protons 'h', so the multiplicity of this peak could not be assigned.

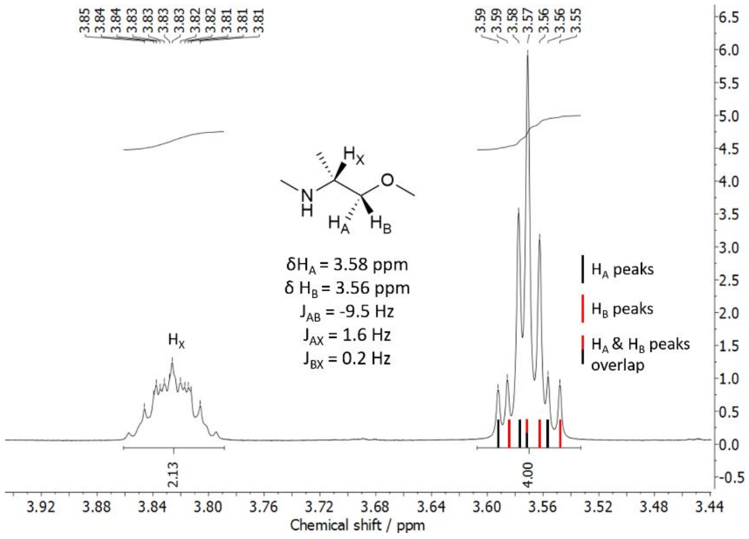

Figure S2. Section of the ^{1}H NMR spectrum of compound **2** showing the ABX system: protons in environments 'i' and 'g'.

Compound **2** was synthesised using a racemic starting material and the product is therefore a mixture of meso and rac diastereoisomers. As a result, protons 'd' produce 4 separate NMR signals: one from each methyl group in the two diastereoisomers. The two central peaks in this signal overlap, so the signal appears as a triplet in both the pure shift and ^{1}H NMR spectra. Four distinct signals are however visible in the carbon spectrum of these methyl groups (Figure S3).

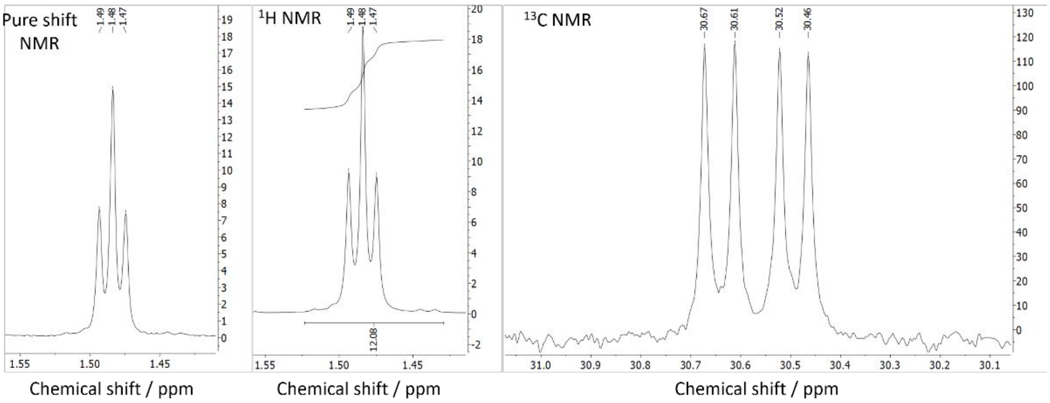

Figure S3. Sections of the pure shift, ^{1}H NMR and ^{13}C NMR spectra of compound **2**, corresponding to protons in environment 'd'.

Synthesis and characterisation of Compound 3

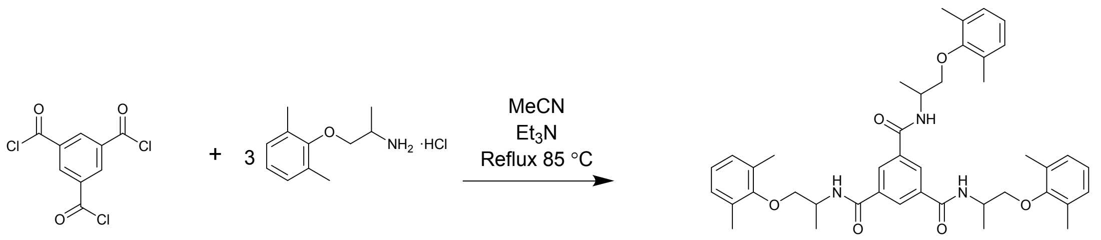

Racemic mexiletine hydrochloride (3.22 g, 13.2 mmol) was suspended in acetonitrile (200 mL) and dissolved upon the addition of excess triethylamine (5 mL). 1,3,5-Benzenetricarbonyl trichloride (1.00 g, 3.77 mmol) was added, the reaction was heated to reflux and stirred for 24 hours. The solvent was removed under vacuum and the crude product was dissolved in DCM (300 mL), washed with water (3 x 200 mL) and recrystallised from THF/diethyl ether to yield compound **3** as a white solid (1.04g, 1.5 mmol, 40 %).

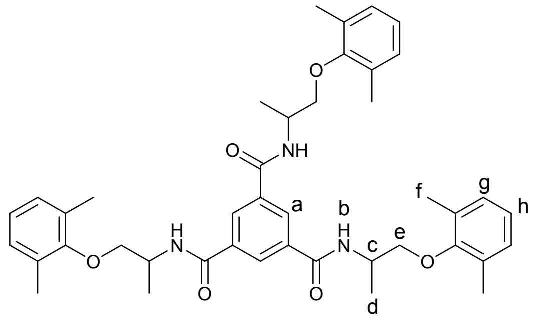

^{1}H NMR (DMSO-d_6, 599 MHz): δ 8.66 (d, *J* = 8.1 Hz, 3H, b), 8.44 (apparent d, *J* = 3.2 Hz, 3H, a)†, 6.97 (d, *J* = 7.5 Hz, 6H, g), 6.87 (t, *J* = 7.5 Hz, 3H, h), 4.47 – 4.37 (m, 3H, c)†, 3.81 – 3.71 (m, 6H, e)†, 2.18 (s, 18H, f), 1.34 (d, *J* = 6.9 Hz, 9H, d).

† See Figure S4 for further details on the assignment of these peaks.

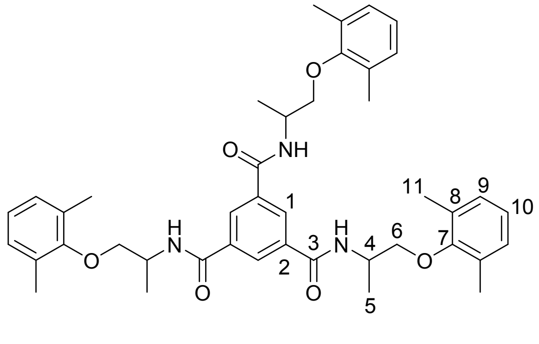

^{13}C{^{1}H} NMR (DMSO-d_6, 151 MHz): δ 165.71 (C3)*, 165.69 (C3)*, 165.67 (C3)*, 155.53 (C8), 135.49 (C2)*, 135.47 (C2)*, 130.74 (C7), 129.20 (C9), 129.16 (C1), 124.18 (C10), 74.37 (C6), 46.08 (C4), 17.61 (C5), 16.33 (C11).

* See Figure S5 for further details of the assignment of these peaks

m/z ESI-MS (MeOH): retention time 4.18 min, 694.4 $[M+H]^+$, 716.4 $[M+Na]^+$, 732.3 $[M+K]^+$, 1409.7 $[2M+Na]^+$, 1425.5 $[2M+K]^+$.

Elemental Analysis: Calc. (%): C 72.70, H 7.41, N 6.06; Found (%): C 72.28, H 7.31, N 5.95.

Compound 3 was synthesised using a racemic starting material and the product is therefore a mixture of eight stereoisomers. Six of these are diastereoisomers that produce unique NMR signals. The presence of multiple diastereoisomers leads to overlapping signals in the pure shift spectrum and a higher apparent multiplicity of signals in the proton spectrum, such as the apparent doublet at 8.44 ppm corresponding to protons ‘a’ (Figure S4). Similarly, overlapping signals from multiple isomers mean that the ABX system, corresponding to protons ‘c’ and ‘e’, cannot be fully resolved. However, the typical pattern of repeating AB quartets can be observed in the multiplet at 3.81-3.71 ppm. The pure shift signal for the two geminal protons, ‘e’, also contains several artefact peaks that are indicate strong coupling between the two protons (Figure S4). The presence of multiple diastereoisomers is also evident in the carbon spectrum, where there are multiple peaks corresponding to carbon environments 2 and 3 (Figure S5).

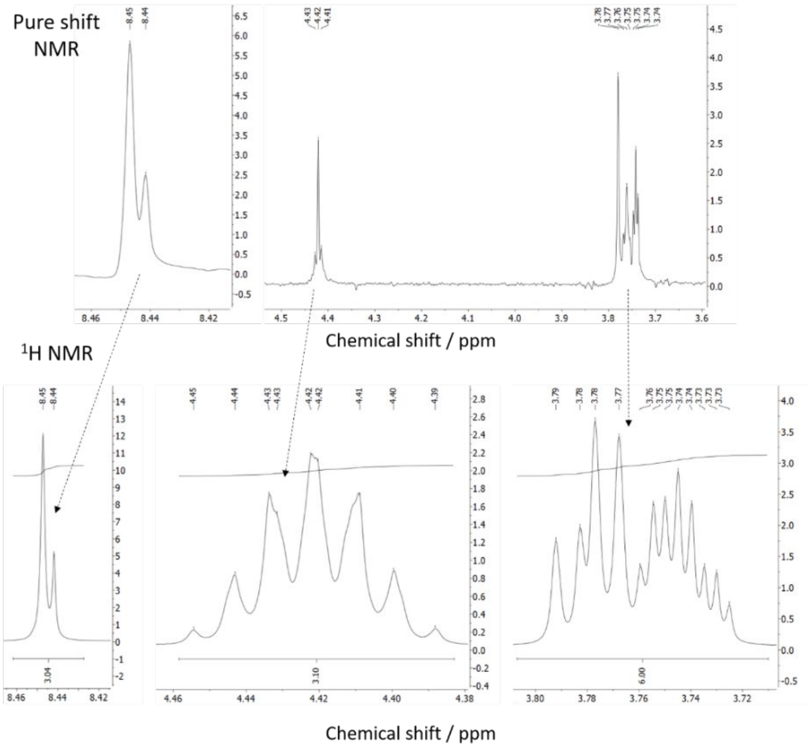

Figure S4. Sections of ^{1}H and pure shift NMR spectra of compound 2 showing the effects of multiple stereoisomers on environments ‘a’, ‘c’ and ‘e’.

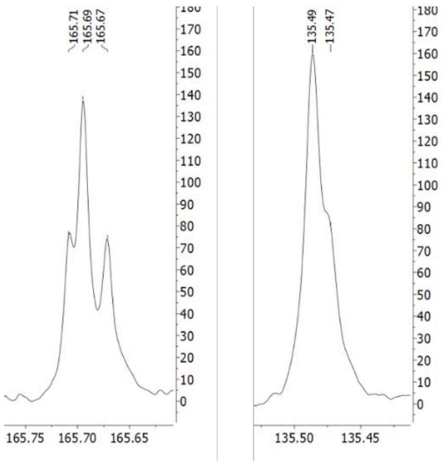

Figure S5. Sections of the ^{13}C NMR spectrum of compound **3** showing signals from environments 2 and 3 in multiple diastereoisomers.

Supplementary Data to the Results & Discussion

Table S1. Gel screening results for Compounds **1**, **2** and **3**. G = gel, PG = partial gel (part of the sample has gelled, but part remains in solution), S = solution, I = insoluble, PPT = precipitate, C = crystals. Solvents with an asterisk were included in the solution-phase polymorph screen, described in our previous work.

Solvent	Compound 1	Compound 2	Compound 3
1,2,4-trichlorobenzene	G	G	G
1,2-dibromoethane	G	G	G
Ethyl methyl ketone, EMK*	G	S	I
1,2-dichlorobenzene	G	G	G
1,3-dichlorobenzene	G	G	G
1,4-dioxane*	G	S	PPT
1-butanol*	G	S	PPT
1-pentanol*	G	S	PPT
1-propanol*	G	S	I
2-butanol*	G	S	I
2-Ethyl pyridine	G	S	G
2-Picoline	G	S	PG + C
2-propanol*	G	S	I
3-chloro-1-propanol	S	S	PPT
3-Picoline	G	S	S
4-Ethyl pyridine	G	S	S
4-Picoline	G	S	S
Acetone*	I	S	I
Acetonitrile*	G	PPT	I
Benzene	PPT	G	I
Benzyl alcohol	G	S	S
Chlorobenzene	G	G	G
Chloroform*	S	S	PPT
Cyclohexane	PPT	PPT	I
Cyclohexanone	G	S	PPT
Cyclopentanone	G	S	S
Dichloromethane*	G	S	PPT
Diethyl ether*	I	I	I
Diethylene glycol	G	S	I
Diisopropyl ether	I	I	I
Dimethylacetamide	G	S	S
DMF*	G	S	S
DMSO*	G	S	S
Ethanol*	G	S	I
Ethyl acetate*	PPT	G	I
Ethylene glycol	PPT	G	I
Ethylene glycol butyl ether	G	S	PPT
Mesitylene	PG	G	I
Methanol*	G	S	I
Nitrobenzene	G	PG	G
Nitromethane*	PG	G	PPT
p-xylene	G	G	S
Pyridine	G	S	PPT
THF*	PG	S	PPT
Toluene*	S	G	I
Water	I	I	PPT
Solvents gelled	35/46	13/46	8/46

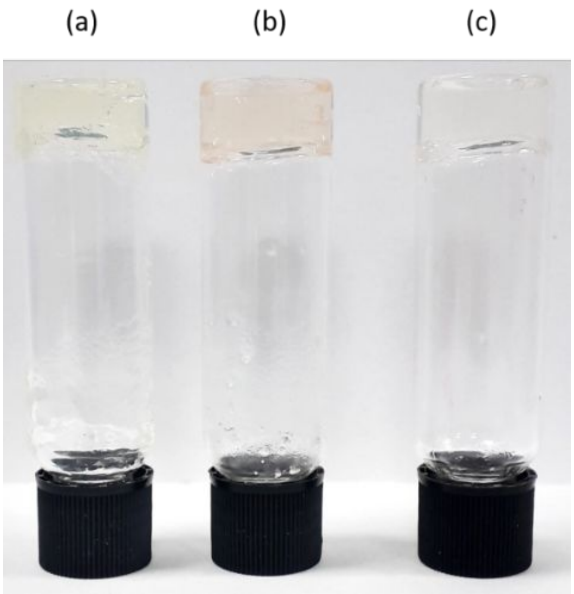

Figure S6. (a) 1 % w/v gel of compound **1** in 1,2,4-trichlorobenzene, (b) 2 % w/v gel of compound **2** in 1,2,4-trichlorobenzene and (c) 2 % w/v gel of compound **3** in 1,2,4-trichlorobenzene.

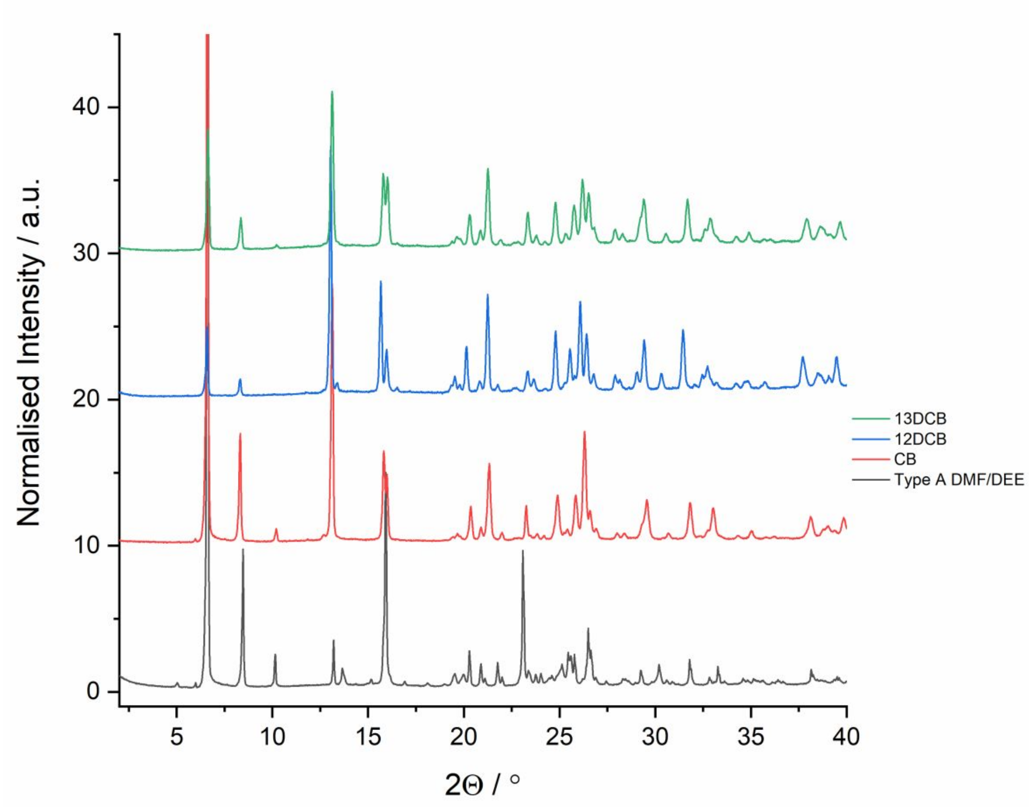

Figure S7. PXRD patterns of the Type A solvates of mexiletine crystallised by slow cooling from chlorobenzene (CB), 1,2-dichlorobenzene (12DCB) and 1,3-dichlorobenzene (13DCB), compared to the Type A diethyl ether solvate crystallised by vapour diffusion of diethyl ether into a mexiletine solution in DMF (DMF/DEE).

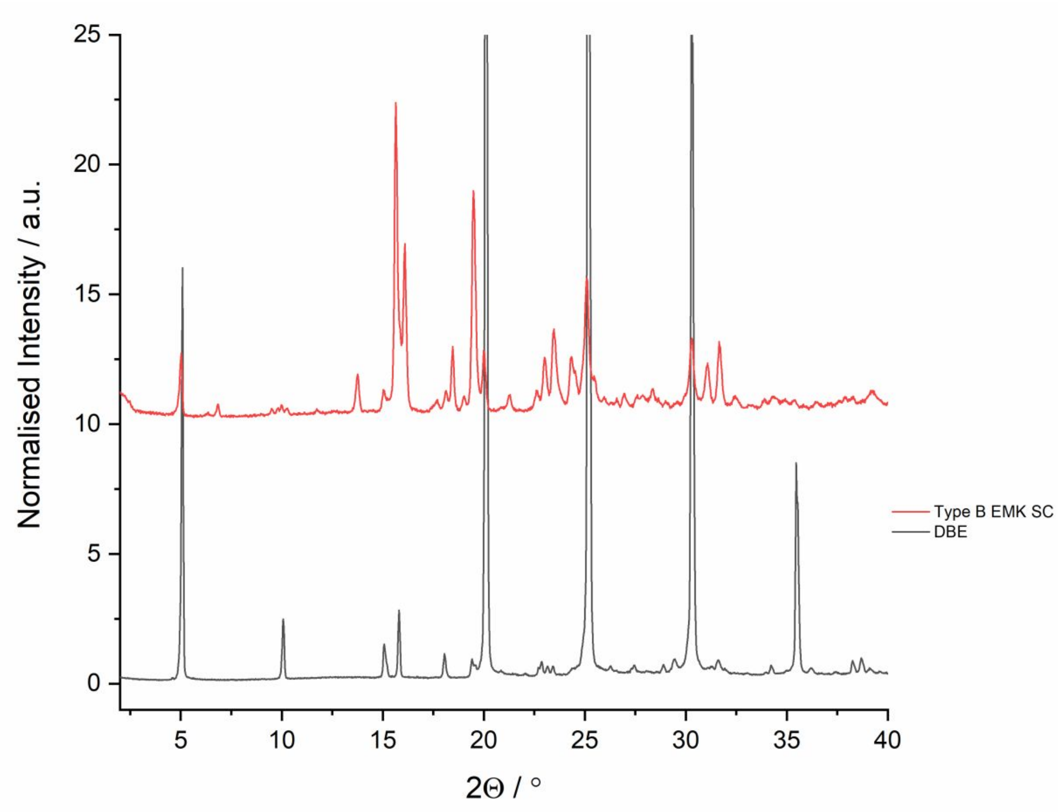

Figure S8. PXRD patterns of the Type B dibromoethane (DBE) and ethyl methyl ketone (EMK) solvates of mexiletine, crystallised by slow cooling.

Table S2. Crystallographic information for the Type C 1,2,4-trichlorobenzene solvate of mexiletine.

Crystallisation conditions	Slow cooling from a supersaturated solution in 1,2,4-trichlorobenzene
Empirical formula	$C_{50}H_{75}Cl_7N_4O_4$
Formula weight	1044.29
Temperature/K	100.0
Crystal system	monoclinic
Space group	$P2_1/c$
a/Å	7.538(3)
b/Å	20.972(9)
c/Å	18.043(8)
α/°	90
β/°	93.725(7)
γ/°	90
Volume/Å^3	2846.0(2)
Z	2
ρ_{calc} g/cm^3	1.219
μ /mm^{-1}	0.362
F(000)	1108.0
Crystal size/mm^3	0.14 × 0.015 × 0.005
Radiation	Synchrotron (λ = 0.6889)
2Θ range for data collection/°	2.89 to 42.986
Index ranges	-8 ≤ h ≤ 8, -22 ≤ k ≤ 22, -19 ≤ l ≤ 19
Reflections collected	20500
Independent reflections	3584 [R_{int} = 0.1746, R_{sigma} = 0.2670]
Data/restraints/parameters	3584/3/277
Goodness-of-fit on F^2	0.867
Final R indexes [I>=2σ (I)]	R_1 = 0.0873, wR_2 = 0.2208
Final R indexes [all data]	R_1 = 0.1593, wR_2 = 0.3023
Largest diff. peak/hole / e Å^{-3}	0.35/-0.20

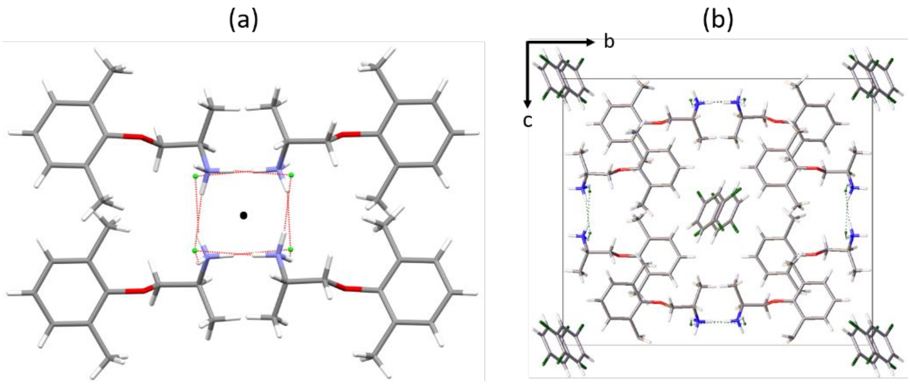

Figure S9. The Type C 1,24-trichlorobenzene solvate, viewed down the *a*-axis, showing (a) the square motif with the inversion centre labelled as a black circle and (b) the disordered solvent situated inside one unit cell of the porous mexiletine framework.

Table S3. Selected crystallographic information for the Type C 1,2,4-trichlorobenzene solvate of mexiletine, compared to the Type A methanol solvate.

	**Type C TCB**	**Type A MeOH**
Space group	$P2_1/c$	*Pbcn*
a/Å	7.538(3)	20.243(7)
b/Å	20.972(9)	18.768(6)
c/Å	18.043(8)	7.550(2)
α/°	90	90
β/°	93.725(7)	90
γ/°	90	90
Volume/Å^3	2846.0(2)	2868.4(15)
Z	2	8
ρ_{calc} g/cm^3	1.219	1.147

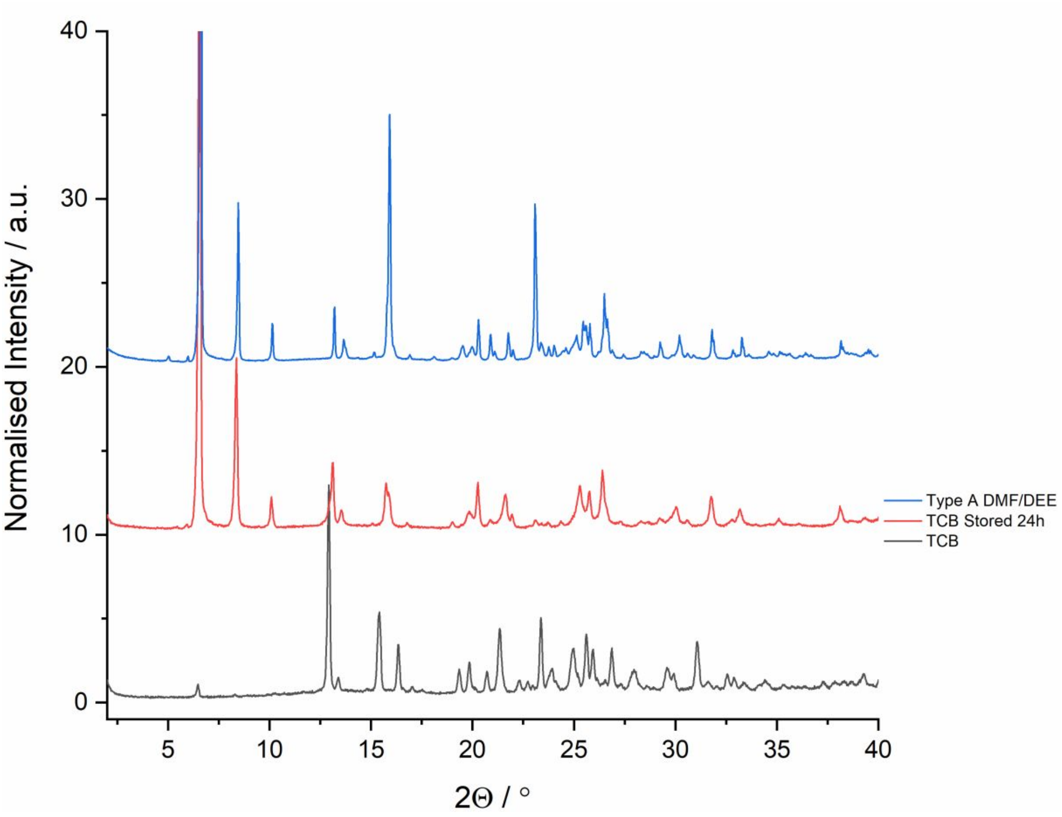

Figure S10. PXRD pattern of the Type C 1,2,4-trichlorobenzene solvate of mexiletine, compared with the same sample after being stored for 24 h, and the Type A diethyl ether solvate crystallised by vapour diffusion of diethyl ether into a saturated solution of mexiletine in DMF.

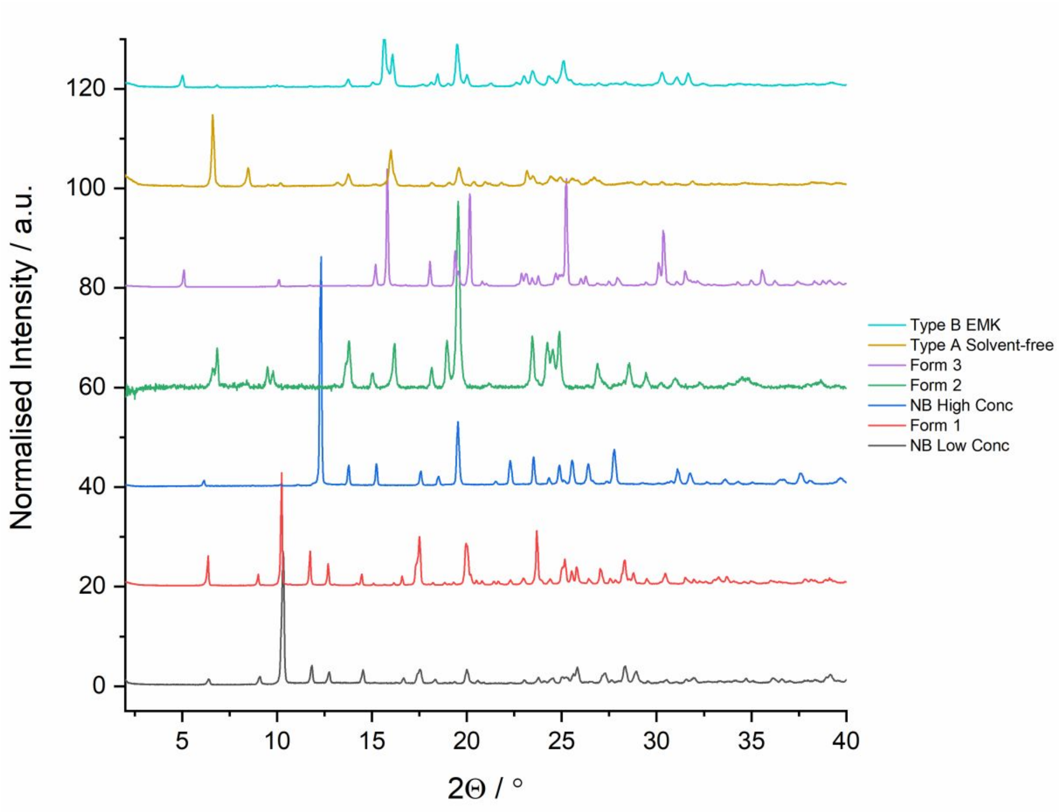

Figure S11. PXRD patterns of the two forms of mexiletine crystallised by slow cooling from nitrobenzene, at a high and low concentration, compared with the five previously known forms. For clarity, one representative example from the Type A and B solvates is shown.

Table S4. Polymorphic outcome of seven slow cooling crystallisations containing various concentrations of mexiletine in nitrobenzene.

Mass of mexiletine / mg	Volume of nitrobenzene / mL	Concentration of mexiletine / % w/v	Polymorph
20	0.05	40	Type D NB Solvate
20	0.1	20	Type D NB Solvate
20	0.15	$13.\dot{3}$	Type D NB Solvate
20	0.2	10	Form 1
20	0.3	$6.\dot{6}$	Form 1
20	0.4	5	Form 1
20	0.5	4	Form1

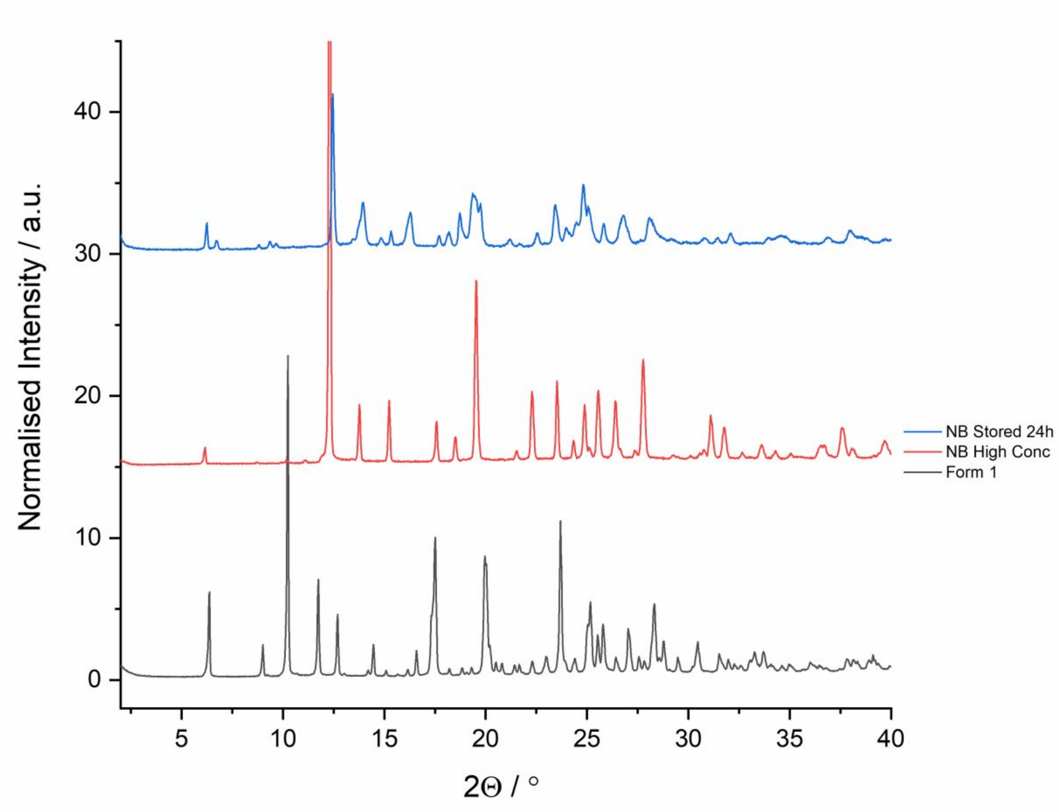

Figure S12. PXRD patterns of the Type D nitrobenzene solvate of mexiletine, crystallised at high concentrations from nitrobenzene, compared to the same sample after being stored for 24 h, and Form 1.

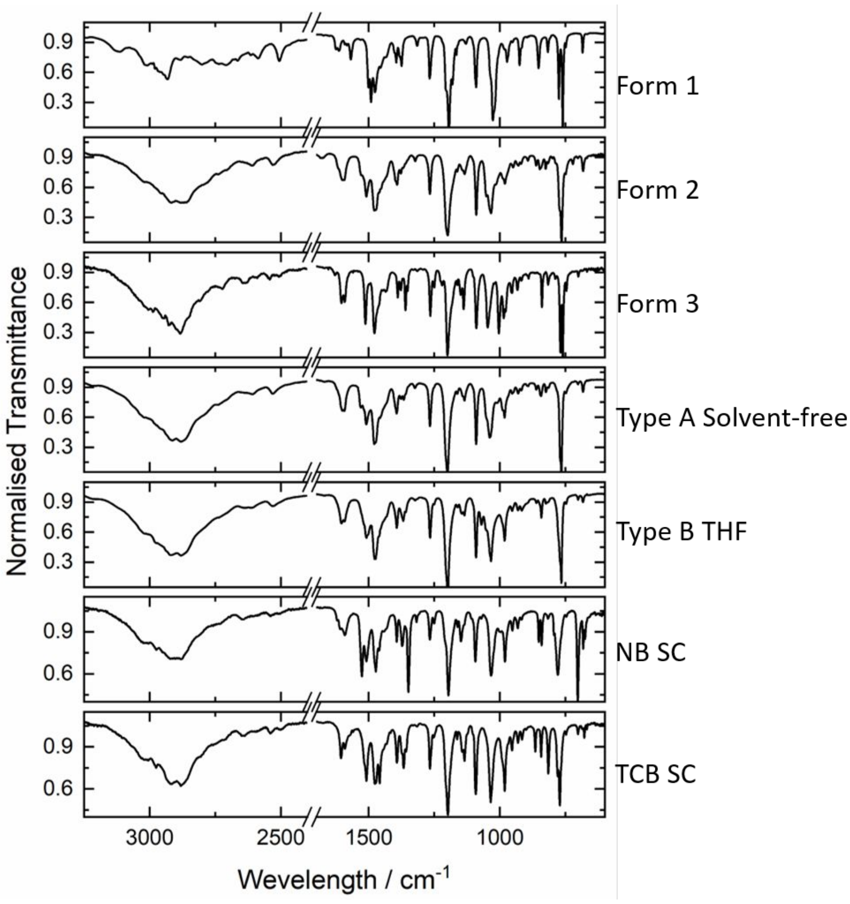

Figure S13. IR spectra of the Type C 1,24-trichlorobenzene solvate and Type D nitrobenzene solvate, both crystallised by slow cooling, compared to the five previously known forms. For clarity, one representative example from the Type A and B solvates is shown.

Table S5. Results of gel-phase crystallisations of mexiletine using compound **3**. G = gel, C = crystals.

Solvent	Gelator Concentration / % w/v	Drug Concentration / % w/v	Gelation Behaviour	Polymorphic Outcome
1,2-Dichlorobenzene	2	5	G+C	Type A
1,3-Dichlorobenzene	2	5	G+C	Type A
Chlorobenzene	2	5	G+C	Type A
1,2-Dibromoethane	2	5	G+C	Type B
1,2,4-Trichlorobenzene	2	5	G+C	Type C TCB
Nitrobenzene	2	5	G+C	Type D NB

Table S6. Results of gel-phase crystallisations of mexiletine using compound **1**. G = gel, C = crystals, P = precipitate, * = gel and solution-phase crystallisations yield different forms.

**Solvent**	**Gelator Concentration / % w/v**	**Drug Concentration / % w/v**	**Gelation Behaviour**	**Polymorphic Outcome**
Nitromethane	2	5	G+PPT	Form 1
Nitromethane	1	2	G+C	Form 1
1-Propanol	1	10	G+C	Form 1
2-Propanol	1	10	G+C	Form 1
1-Butanol	1	10	G+C	Form 1
2-Butanol	1	10	G+C	Form 1
Amyl Alcohol	1	10	G+C	Form 1
Acetonitrile	1	2	C	Form 1
Acetonitrile	2	5	C	Form 3
DCM	1	2	G+PPT	Type A
DCM	2	5	G+C	Type A
THF	1	2	G+C	Type B
THF	0.5	1	G+C	Type B
1,4-Dioxane	1	10	G+C	Type B
1,4-Dioxane	0.5	10	G+C	Type B
EMK	1	2	G+C	Type B
EMK	0.5	1	G+C	Form 3*
DMF	2	5	G+PPT	Type A*
DMF	1	10	PPT	Type A*
DMSO	2	5	G+PPT	Type A*
1,2-Dichlorobenzene	1	5	G+C	Type A
1,3-Dichlorobenzene	1	5	G+C	Type A
Chlorobenzene	1	5	G+PPT	Type A
1,2-Dibromoethane	1	5	G+C	Type B
1,2,4-Trichlorobenzene	1	5	G+C	Type C TCB
Nitrobenzene	1	5	G+C	Type D NB

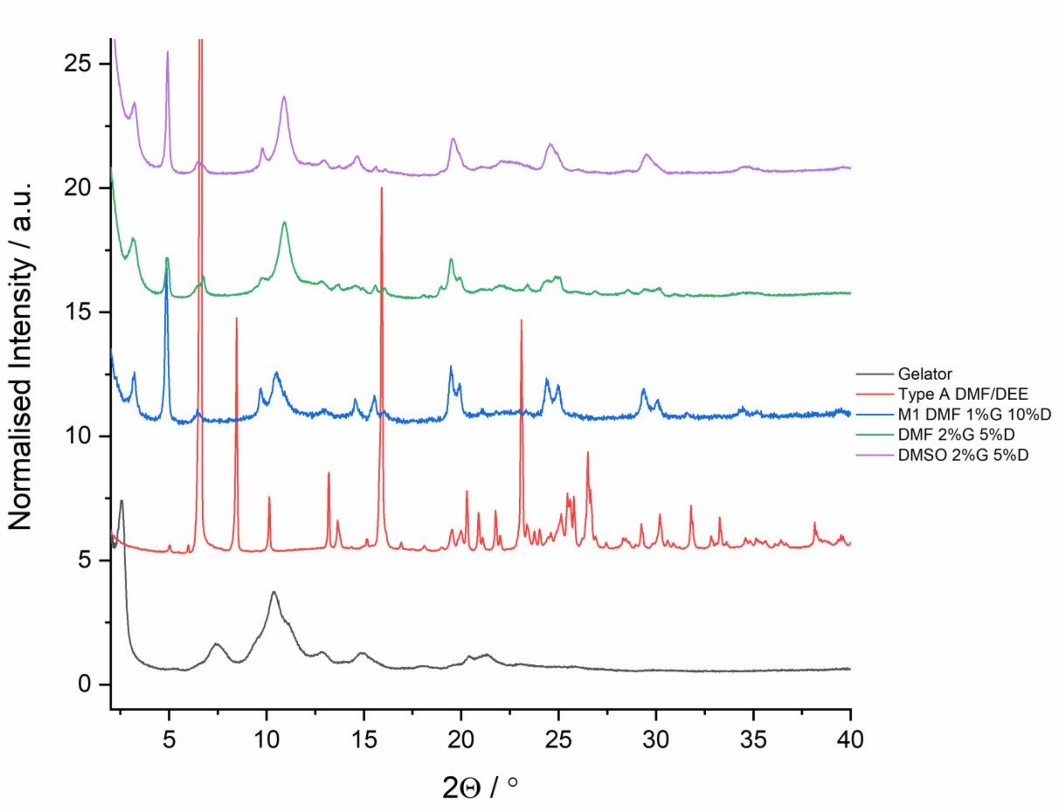

Figure S14. PXRD patterns of the mexiletine solid forms crystallised from three DMF and DMSO gels of compound **1**, compared to the gelator, and the Type A diethyl ether solvate crystallised by vapour diffusion of diethyl ether into DMF.

Table S7. Results of gel-phase crystallisations of mexiletine using compound **2**. G = gel, C = crystals, P = precipitate, * = gel and solution-phase crystallisations yield different forms, Type A′ = the contents of the channels differs between the Type A solvates crystallised from solutions and gels.

**Solvent**	**Gelator Concentration / % w/v**	**Drug Concentration / % w/v**	**Gelation Behaviour**	**Polymorphic Outcome**
Nitromethane	2	5	C	Form 1
Nitromethane	2	2	G+C	Form 1
Nitromethane	2	2	C	Form 1
Nitromethane	2	1	Weak G+C	Form 1
Nitromethane	2	1	C	Form 1
Toluene	2	5	C	Type A Tol
Toluene	2	2	C	Type A Tol
Toluene	2	2	G+C	Type A Tol
Toluene	2	1	C	Type A Tol
Toluene	2	1	PG+C	Type A′*
Ethyl Acetate	2	5	C	Type A*
Ethyl Acetate	2	2	C	Type A*
Ethyl Acetate	2	2	C	Form 1
Ethyl Acetate	2	2	G+C	Form 1
Ethyl Acetate	2	1	C	Form 1
Ethyl Acetate	2	1	C	Type A′*
1,2-Dichlorobenzene	2	5	G+C	Type A
1,3-Dichlorobenzene	2	5	G+C	Type A
Chlorobenzene	2	5	G+C	Type A
1,2-Dibromoethane	2	5	G+C	Form 2*
1,2,4-Trichlorobenzene	2	5	G+C	Type C TCB
Nitrobenzene	2	5	G+C	Type D NB

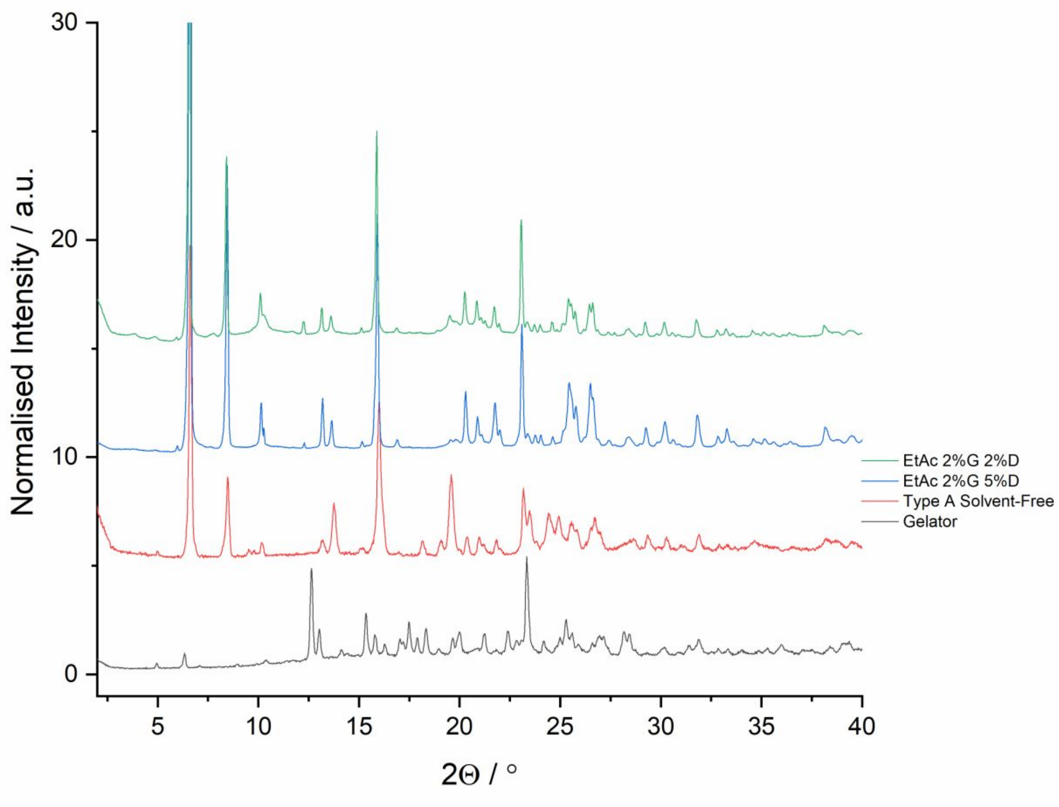

Figure S15. PXRD patterns of mexiletine crystallised from solutions in ethyl acetate, at concentrations of 2 % w/v gelator and 2 or 5 % w/v drug, compared to the Type A solvent-free form, and the gelator.

1. Dolomanov, O. V.; Bourhis, L. J.; Gildea, R. J.; Howard, J. A. K.; Puschmann, H., OLEX2: a complete structure solution, refinement and analysis program. *J. Appl. Crystallogr.* **2009**, *42*, 339-341.
2. Sheldrick, G. M., A short history of SHELX. *Acta Crystallogr. Sect. A* **2008**, *64*, 112-122.
3. Knolker, H. J.; Braxmeier, T.; Schlechtingen, G., A Novel Method For The Synthesis Of Isocyanates Under Mild Conditions. *Angew. Chem., Int. Ed.* **1995**, *34*, 2497-2500.
